# Supplementary figures and images for: The Cross-Regulation Between Set1, Clr4, and Lsd1/2 in Schizosaccharomyces pombe
Source: PLoS Genet. 2024 Jan 5;20(1):e1011107. doi: 10.1371/journal.pgen.1011107 (PMC10795994; doi:10.1371/journal.pgen.1011107)

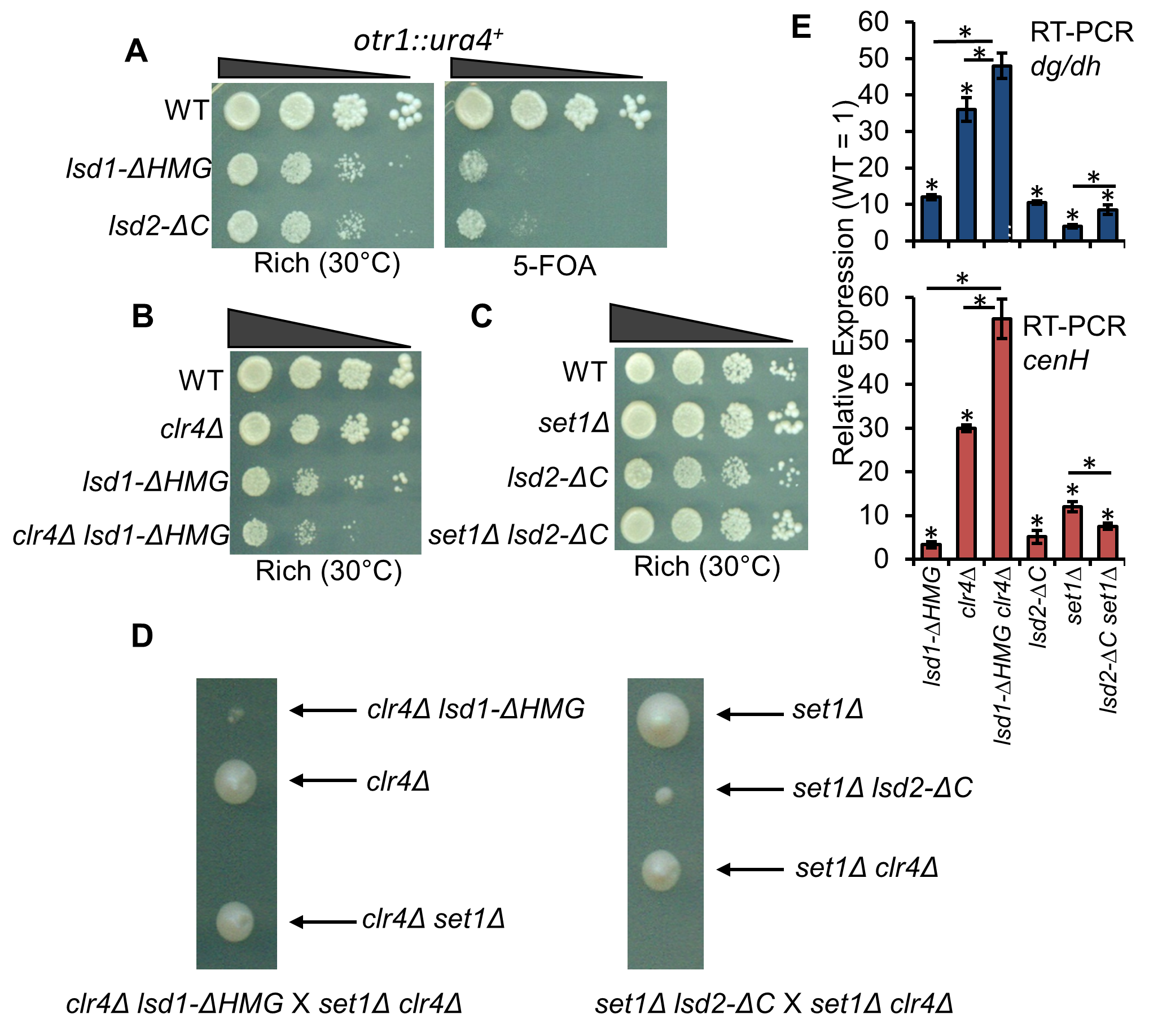

Supplement: S1 Fig — (A) Serial dilution assays reveal growth defects in lsd1-ΔHMG and lsd2-ΔC mutants on rich media (YEA) at the permissive temperature (30°C), along with silencing defects at the outer centromeric repeat (otr1::ura4+) on 5-FOA. (B-C) Serial dilution assays demonstrate viable genetic interactions between lsd1-ΔHMG and clr4Δ (B) and between lsd2-ΔC and set1Δ (C) on rich media (YEA) at the permissive temperature (30°C). (D) A representative tetrad yielded from genetic crosses between clr4Δ set1Δ and lsd1-ΔHMG clr4Δ (left), or lsd2-ΔC set1Δ (right), reveals the lethality of lsd1-ΔHMG set1Δ clr4Δ and lsd2-ΔC set1Δ clr4Δ triple mutants. (E) qRT-PCR analysis of peri-centromeric dg/dh repeats and cenH (mating type locus) demonstrates heterochromatin silencing defects in lsd1-ΔHMG clr4Δ and lsd2-ΔC set1Δ double mutants, with normalization to wild-type (WT = 1). Asterisks denote significance (p ≤ 0.05) determined by the Student’s t-test. Horizontal lines indicate significance between single mutants and double mutants. Error bars represent the standard error of the mean (s.e.m.). (TIF) [file pgen.1011107.s001.tif]

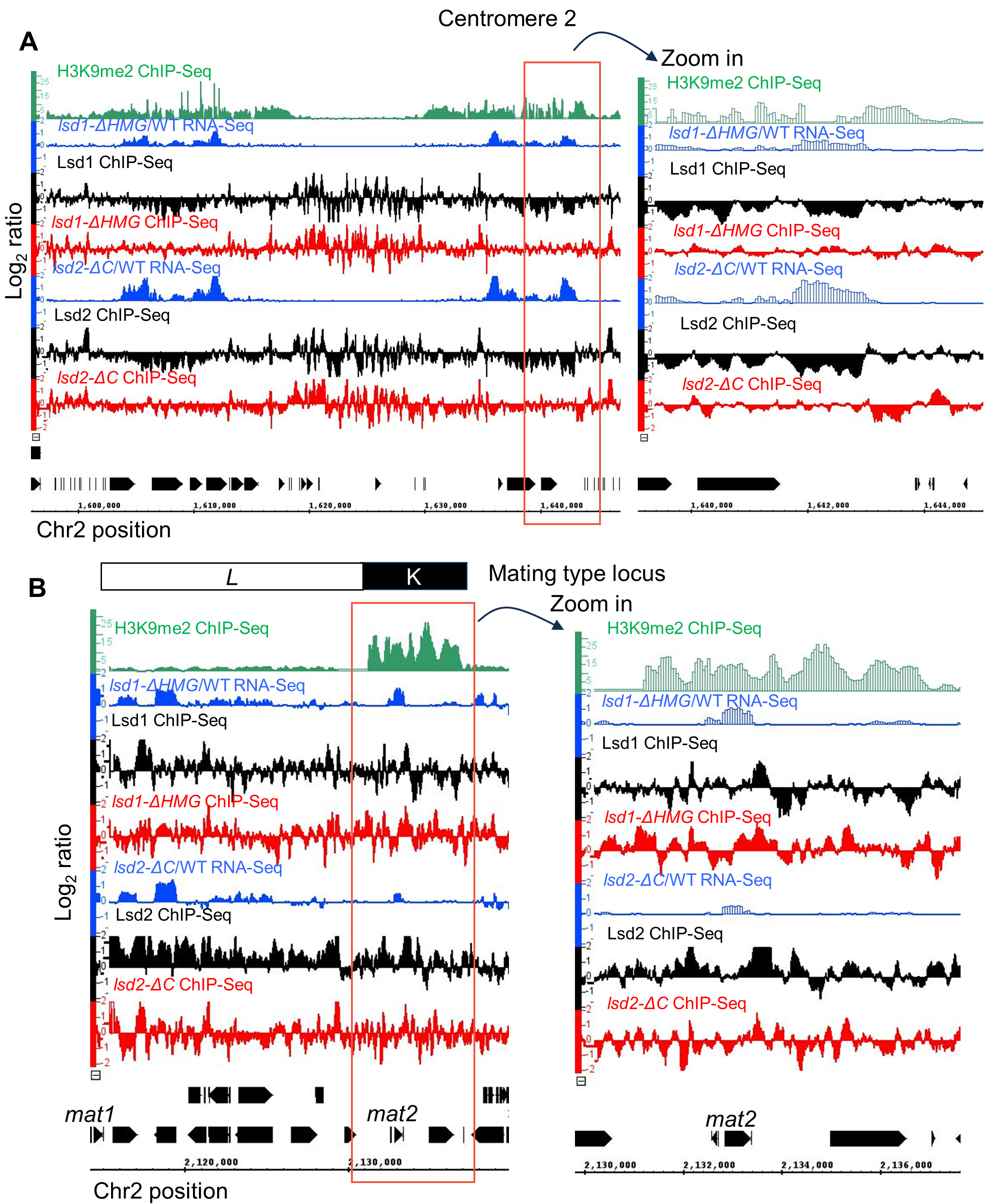

Supplement: S2 Fig — (A-B) Loss of silencing at the peri-centromeric region (A) and the mating type locus (B) in lsd1-ΔHMG and lsd2-ΔC mutants compared to wild-type (WT) using RNA-seq data are illustrated in blue. Overlayed are wild-type Lsd1 or Lsd2 ChIP-Seq data in black and lsd1-ΔHMG or lsd2-ΔC ChIP-Seq data in red. The chromosome position is shown on the X-axis, and the Y-axis represents the standardized Log2 ratios for Lsd1/2 RNA-Seq and ChIP-Seq (range from -2 to 2) and H3K9me2 ChIP-Seq (range from 0 to 30). H3K9me2 ChIP-Seq showing the heterochromatic regions are highlighted in green. Zoomed-in regions of the red box on the left panel are displayed on the right (A-B). Figures were generated using the Integrated Genome Browser (IGB). (TIF) [file pgen.1011107.s002.tif]

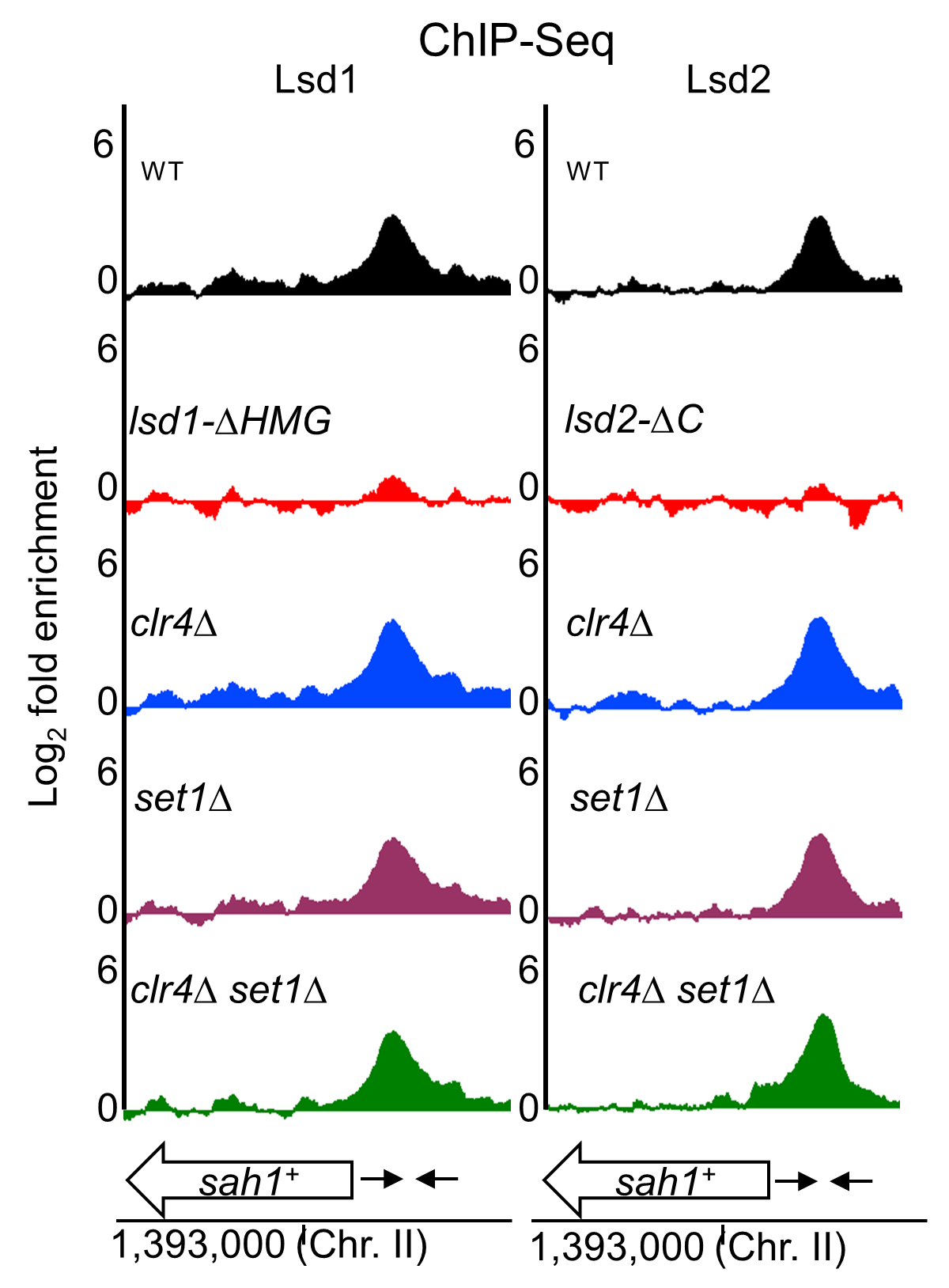

Supplement: S3 Fig — ChIP-Seq peaks showcase the localization of Lsd1/2 at the sah1+ promoter region, with sah1+ schematic denoted on the X-axis. Arrows besides the sah1+ gene pinpoint the oligo positions mentioned in Fig 1H. The Y-axis displays standardized (range from 0 to 6) Log2 fold enrichments to untagged control. This figure was generated using the Integrated Genome Browser (IGB). (TIF) [file pgen.1011107.s003.tif]

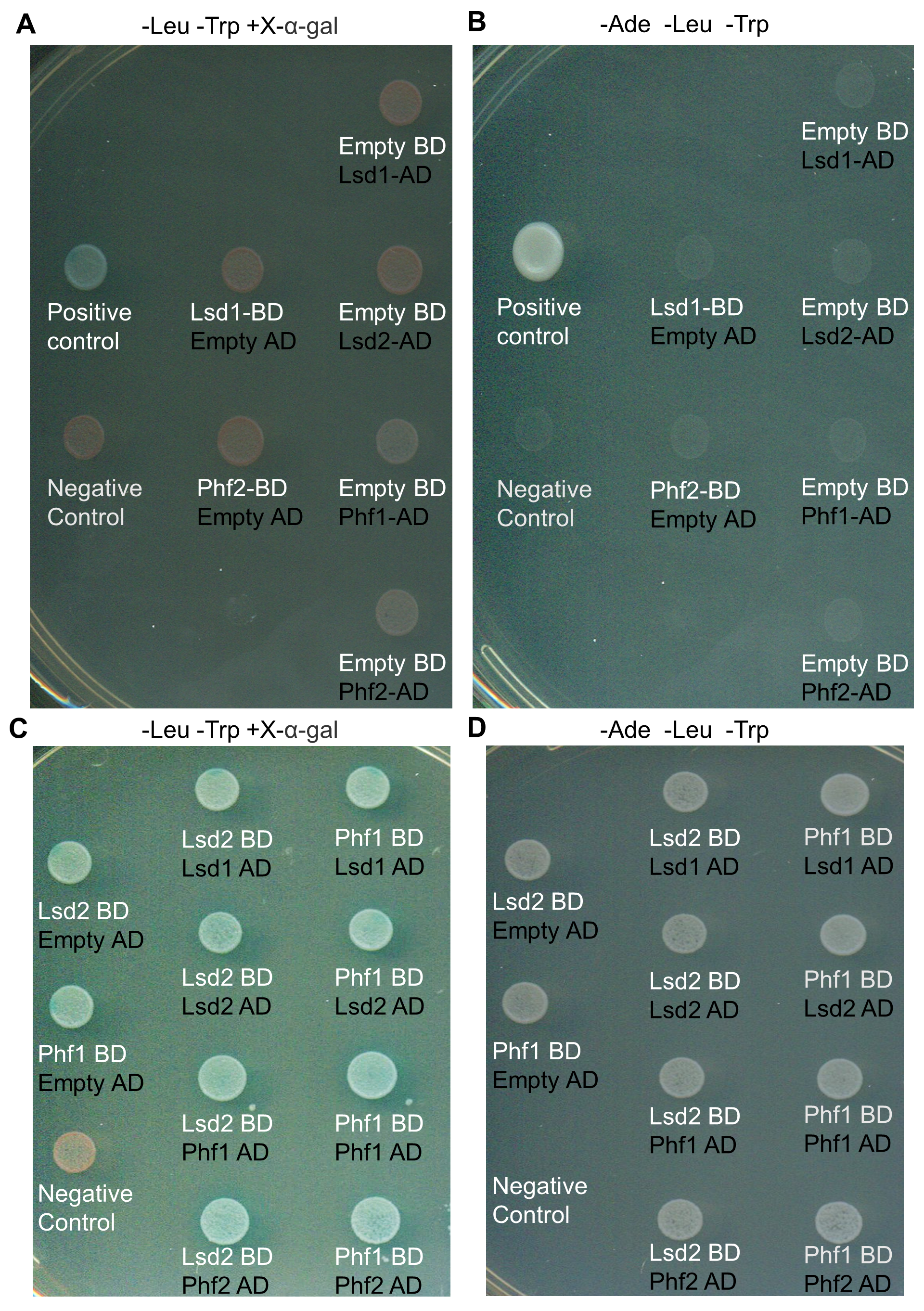

Supplement: S4 Fig — (A-B) When combined with an empty vector as a negative control in the yeast two-hybrid system, Lsd1-AD, Lsd1-BD, Lsd2-AD, Phf2-BD, Phf1-AD, and Phf2-AD do not induce reporter gene expression. (C-D) Lsd2-BD and Phf1-BD autonomously triggered the expression of reporter genes when combined with an empty AD vector. These colonies exhibited growth on two selective plates: (A & C) medium lacking leucine and tryptophan but containing X-α-gal (-Leu -Trp +X-α-gal) and (B & D) medium without adenine, leucine, and tryptophan (-Ade -Leu -Trp). (TIF) [file pgen.1011107.s004.tif]

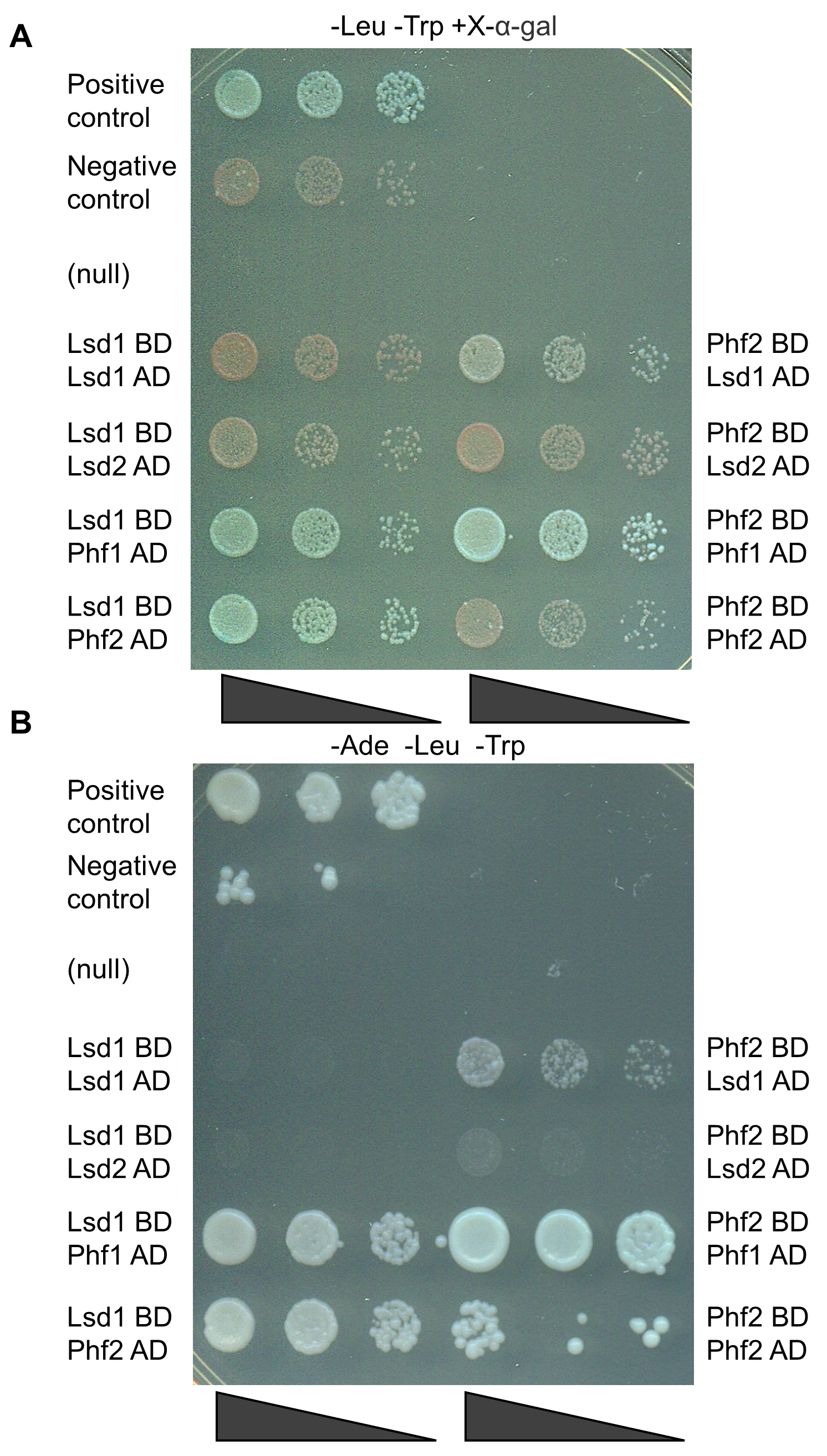

Supplement: S5 Fig — (A-B) Serial dilution assays show direct interactions between Lsd1 with Phf1 and Phf2. In contrast, Lsd2 does not engage in direct interactions with either Lsd1 or Phf2. Colonies on two selective plates confirm these interactions: (A) medium lacking leucine and tryptophan but supplemented with X-α-gal (-Leu -Trp +X-α-gal) and (B) medium without adenine, leucine, and tryptophan (-Ade -Leu -Trp). (TIF) [file pgen.1011107.s005.tif]

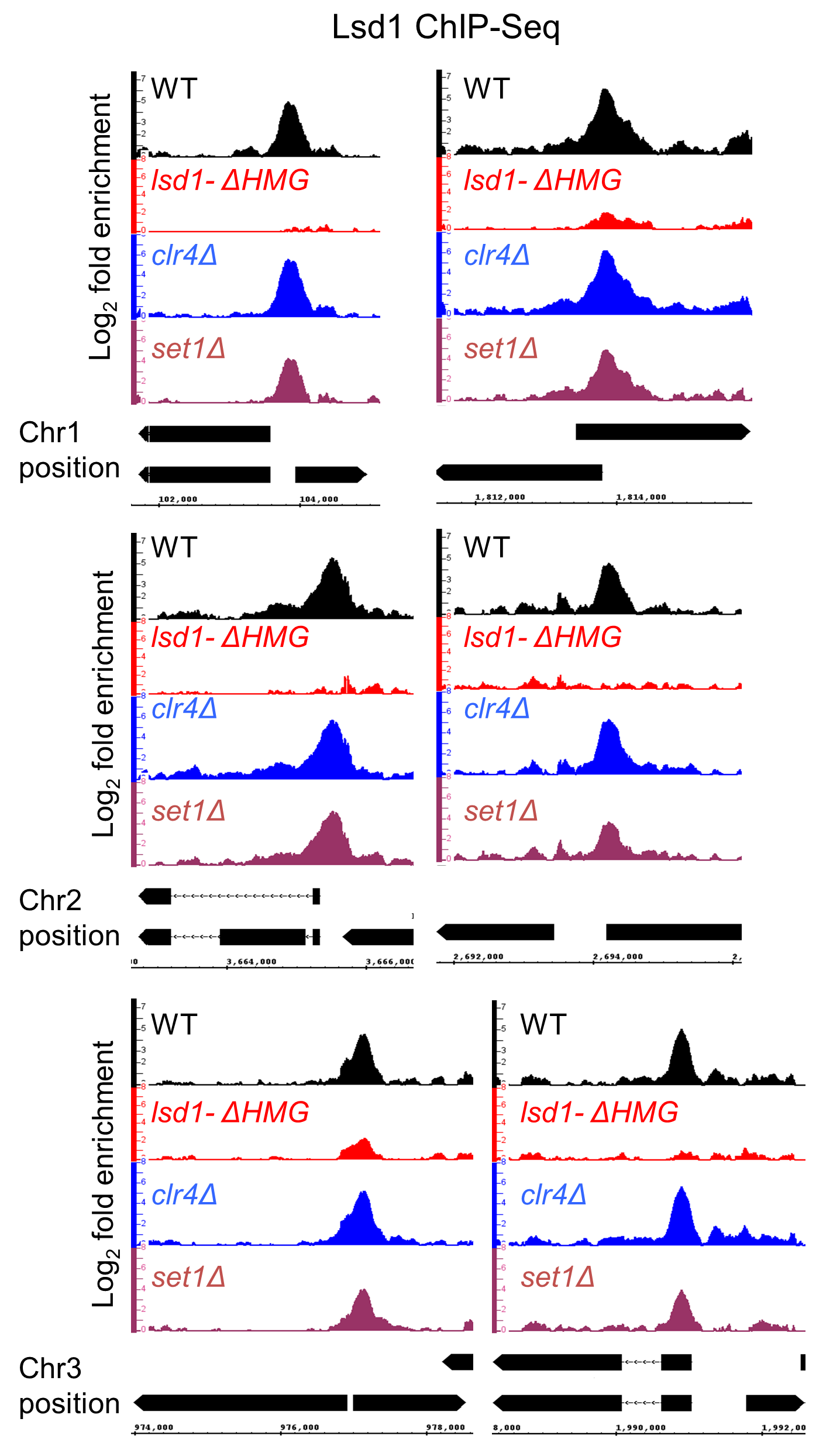

Supplement: S6 Fig — ChIP-Seq peaks for Lsd1 at specified genetic backgrounds reveal its localization in two distinct loci on each chromosome. The X-axis shows the genomic position on each indicated chromosome. The Y-axis represents standardized Log2 fold enrichments (range from 0 to 8) compared to the untagged control. This figure was made using the Integrated Genome Browser (IGB). (TIF) [file pgen.1011107.s006.tif]

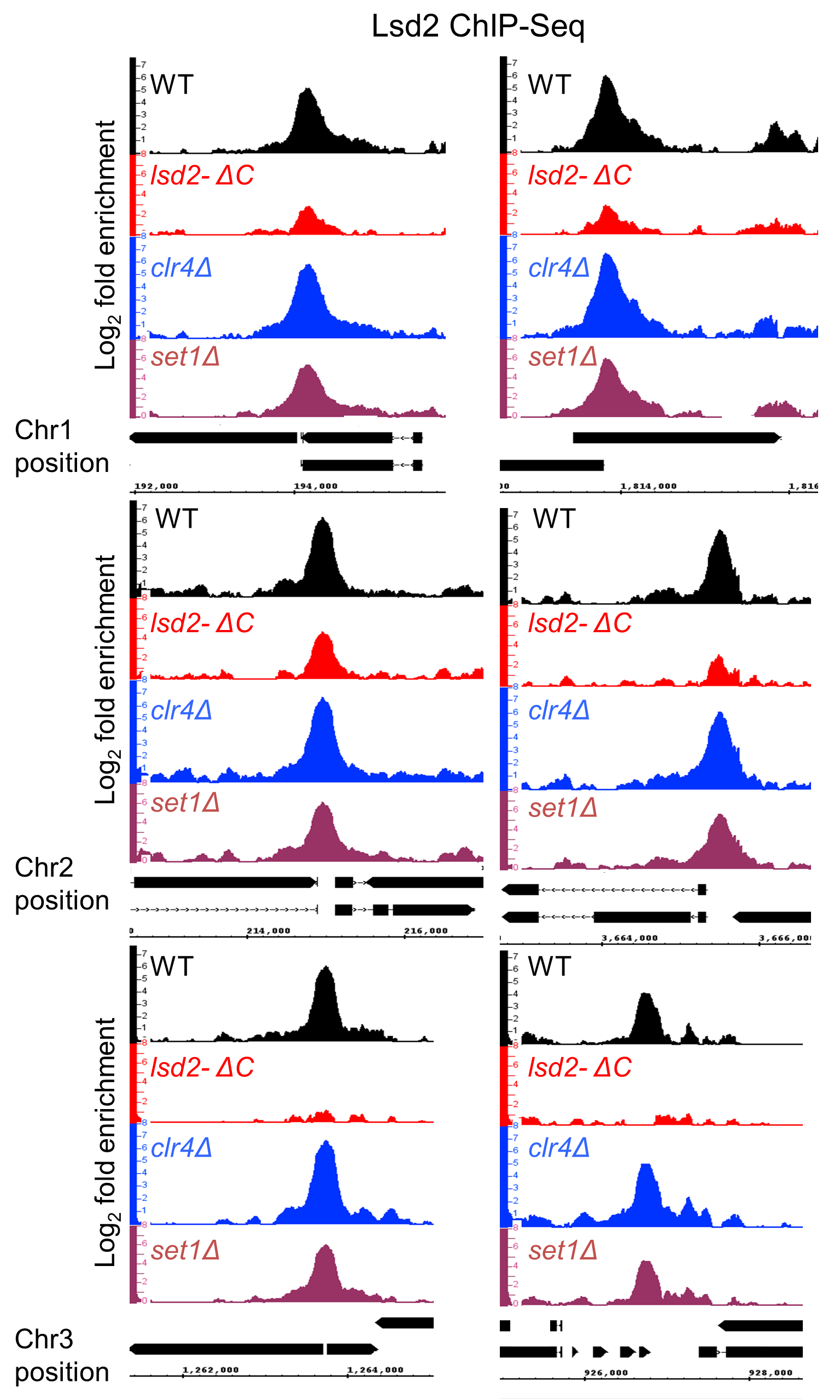

Supplement: S7 Fig — The ChIP-Seq peaks for Lsd2 in indicated genetic contexts highlight its positioning at two specific loci on each chromosome. The X-axis indicates the genomic position on each indicated chromosome, and the Y-axis represents standardized Log2 fold enrichments (range from 0 to 8) relative to the untagged control. This figure was generated in the Integrated Genome Browser (IGB). (TIF) [file pgen.1011107.s007.tif]

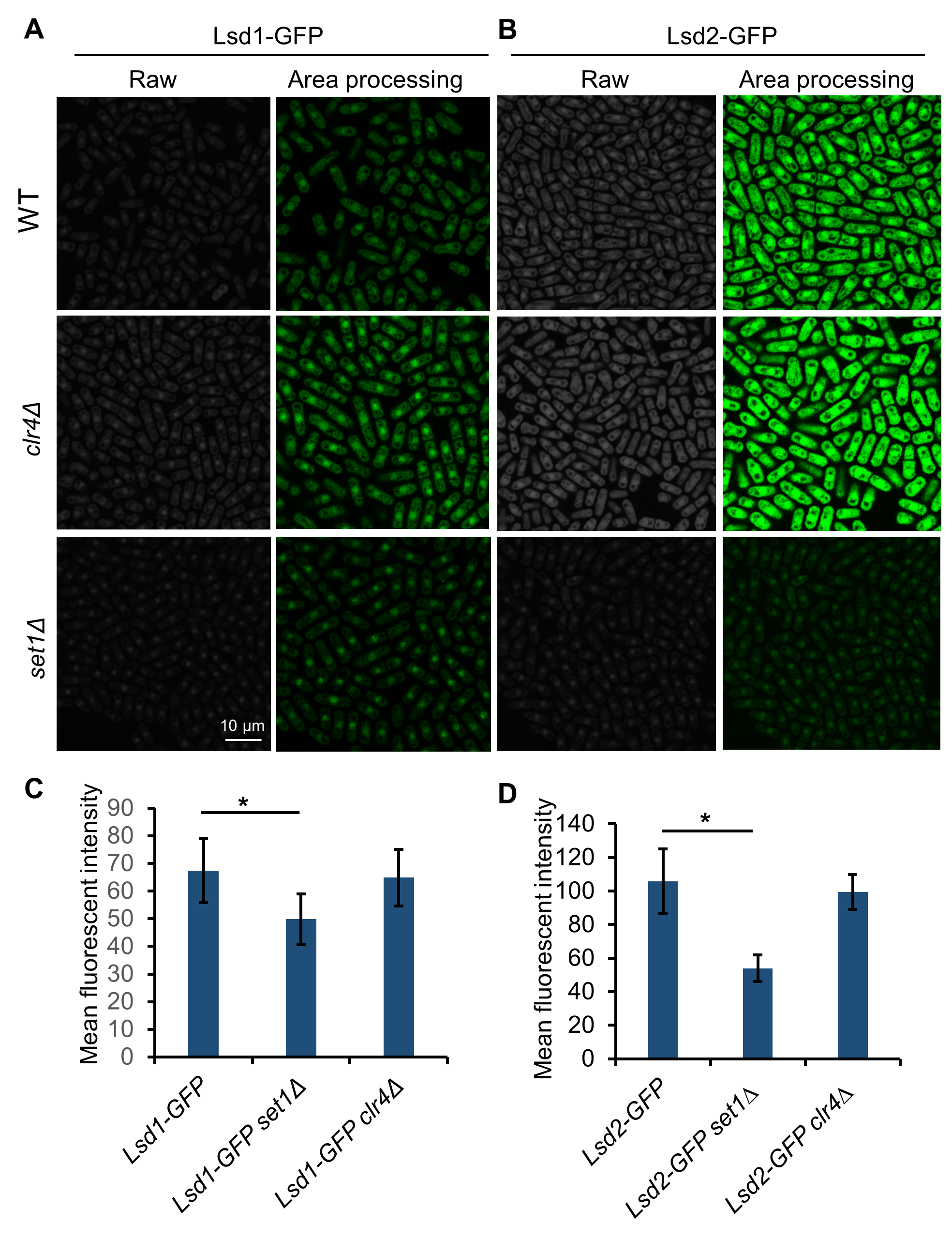

Supplement: S8 Fig — (A-B) Lsd1-GFP (A) and Lsd2-GFP (B) in indicated genetic backgrounds were imaged using a Zeiss LSM880 confocal microscope. (C-D) Quantification of approximately 100 cells per raw image was conducted using Image J software. Lsd1-GFP (C); Lsd2-GFP (D). Asterisks denote significance (p ≤ 0.05) determined by the Student’s t-test. Horizontal lines indicate significance between wild-type and mutants. Error bars represent the standard error of the mean (s.e.m.). (TIF) [file pgen.1011107.s008.tif]

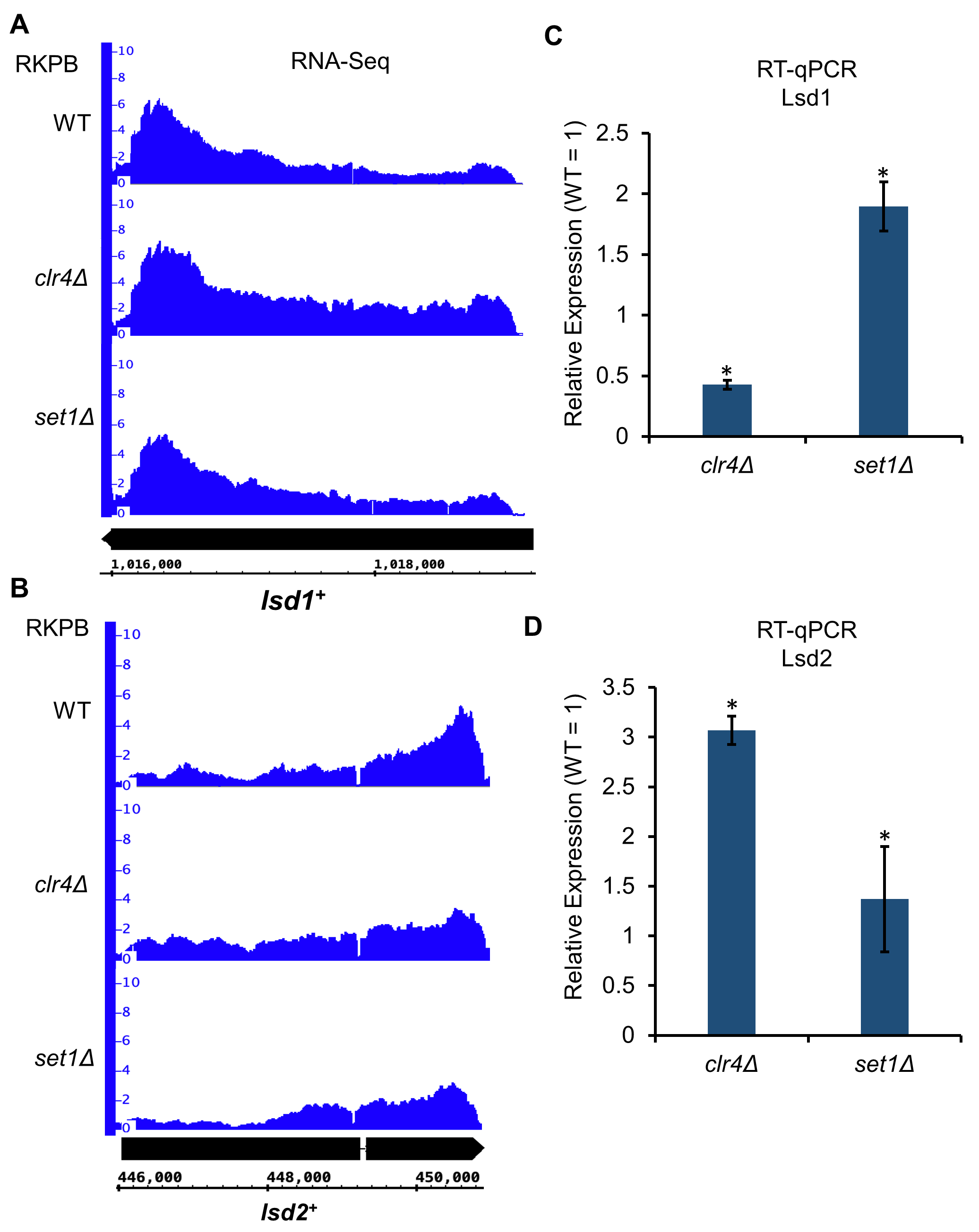

Supplement: S9 Fig — (A -B) RNA-Seq analysis compares normalized RNA-Seq reads (RPKB) aligned with the genomic locus of Lsd1 (A) and Lsd2 (B) in clr4Δ or set1Δ backgrounds to those in the wild type. The data reveals that the loss of Clr4 or Set1 has a minimal impact on the overall mRNA levels of lsd1+ and lsd2+. The graphical representation was generated using the Integrated Genome Browser (IGB). (C-D) Quantitative RT-PCR analysis of lsd1+ (C) and lsd2+ (D) mRNA levels reinforce the observation that changes in mRNA levels in clr4Δ and set1Δ do not align with variations in Lsd1 and Lsd2 protein levels when normalized to wild-type (WT = 1). Asterisks indicate statistical significance (p ≤ 0.05) as determined by the Student’s t-test when comparing the indicated samples with WT values. Error bars represent the standard error of the mean (s.e.m.). (TIF) [file pgen.1011107.s009.tif]

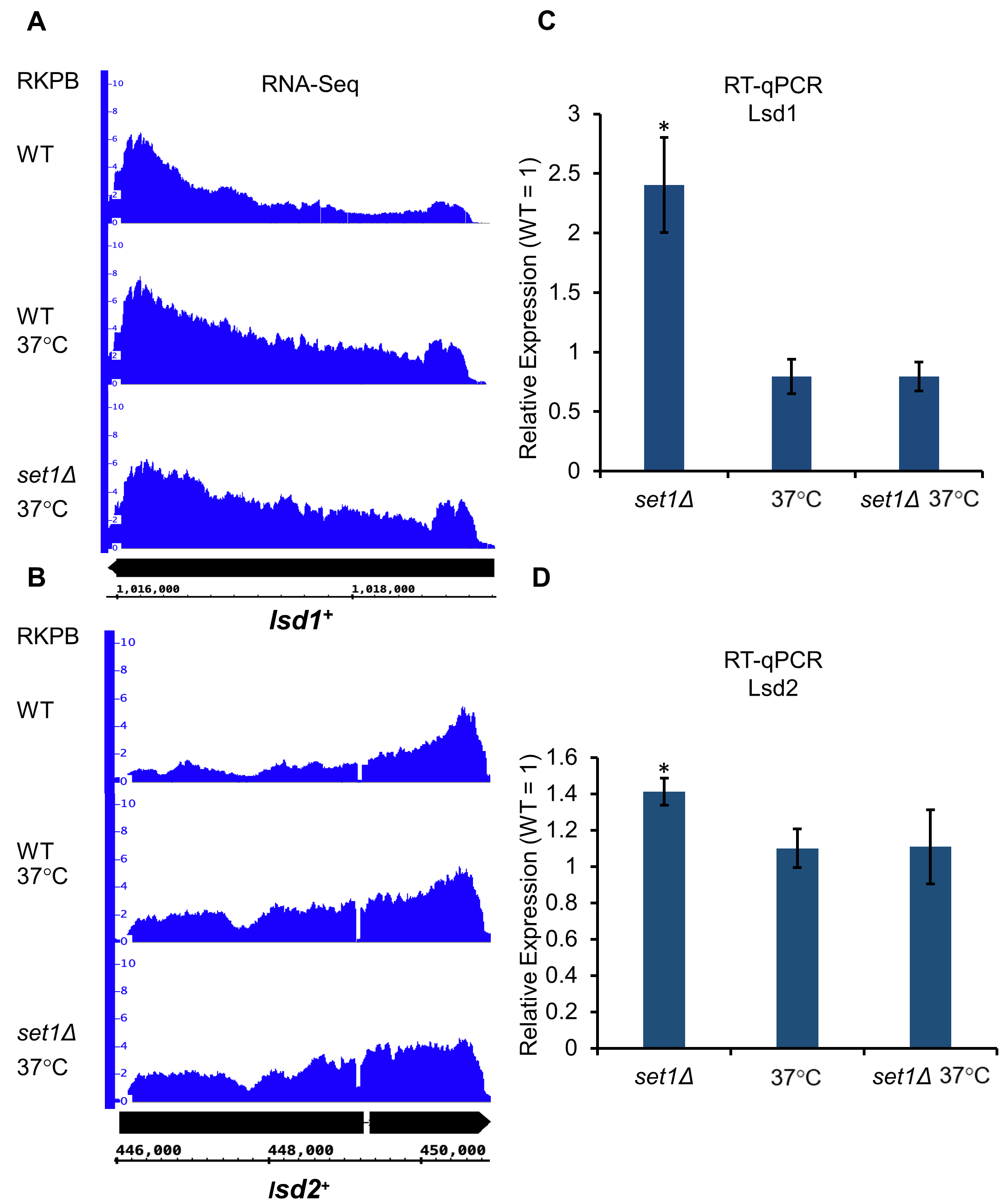

Supplement: S10 Fig — (A-B) We analyzed normalized RNA-Seq reads (RPKB) aligned to the genomic loci of lsd1+ (A) and lsd2+ (B) in wild-type cells at both 30°C and 37°C. Additionally, we assessed the mRNA levels of lsd1+ and lsd2+ in a set1Δ background at 37°C. Graphs were made using the Integrated Genome Browser (IGB). (C-D) qRT-PCR analysis of lsd1+ mRNA levels (C) and lsd2+ mRNA levels (D) demonstrate the impact of heat stress on Lsd1/2 transcription, with or without Set1, normalized to wild-type (WT = 1). Statistical significance (p ≤ 0.05) is indicated by asterisks, determined by the Student’s t-test when comparing the indicated samples with WT values. Error bars represent the standard error of the mean (s.e.m.). (TIF) [file pgen.1011107.s010.tif]
