# Supplementary material for: The Cross-Regulation Between Set1, Clr4, and Lsd1/2 in Schizosaccharomyces pombe
Source: PLoS Genet. 2024 Jan 5;20(1):e1011107. doi: 10.1371/journal.pgen.1011107 (PMC10795994; doi:10.1371/journal.pgen.1011107)
Supplement: S3 Table — (PDF) [file pgen.1011107.s013.pdf]

S3 Table. List of strains used in this study (1 of 10)

**Strains (*S. pombe*)**	***mat***	***leu1***	***ade6***	***his2***	***ura4***	**Epigenetic Reporter**	**Mutation**
**SPWF114**	h+	*leu1-32*	*210*	*plus*	*DS/E*	*otr1R(Sph1)::ura4+*	-
**SPFM115**	Mat1 Msmt0	*leu1-32*	*216*	*his2*	*DS/E*	*otr1R(Sph1)::ura4+*	-
**SPFM329**	Mat1 Msmt0	*leu1-32*	*216*	*his2*	*DS/E*	*otr1R(Sph1)::ura4+*	*Lsd1-FTP::NatN2*
**SPFM370**	Mat1 Msmt0	*leu1-32*	*210 or 216*	*his2*	*DS/E*	*otr1R(Sph1)::ura4+*	*Lsd2-FTP::NatN2*
**SPHL203**	h+	*leu1-32*	*216*	*plus*	*DS/E*	*otr1R(Sph1)::ura4+*	*Lsd1-FTP::NatN2*
**SPHL135**	h+	*leu1-32*	*210*	*plus*	*DS/E*	*otr1R(Sph1)::ura4+?*	*Lsd2-FTP::NatN2*
**SPJT437**	Mat1 Msmt0	*leu1-32*	*210*	*his2*	*DS/E*	*otr1R(Sph1)::ura4+*	*lsd1-ΔHMG-FTP::NatN2*
**SPJT441**	Mat1 Msmt0	*leu1-32*	*216*	*his2*	*DS/E*	*otr1R(Sph1)::ura4+*	*lsd2-ΔC-FTP::NatN2*
**SPWF73**	diploid	*leu1-32//leu1-32*	*210//216*	*his2//plus*	*DS/E//DS/E*	*otr1R(Sph1)::ura4+//otr1R(Sph1)::ura4+*	-
**SPJT475**	h90	*leu1-32*	*210 or 216*	*his2*	*DS/E or ura4+*	*otr1R(Sph1)::ura4+?*	*lsd1-ΔHMG-FTP::NatN2*
**SPJT476**	h90	*leu1-32*	*210 or 216*	*his2*	*DS/E*	*otr1R(Sph1)::ura4+?*	*lsd2-ΔC-FTP::NatN2*
**SPFM401**	h+	*leu1-32*	*216*	*plus*	*DS/E*	*otr1R(Sph1)::ura4+*	*Phf1-myc::kanMX*
**SPJC144**	Mat1 Msmt0	*leu1-32*	*210 or 216*	*his2*	*DS/E*	*otr1R(Sph1)::ura4+?*	*lsd1-ΔHMG-FTP::NatN2, Phf1-myc::kanMX*
**SPJC129**	Mat1 Msmt0	*leu1-32*	*210 or 216*	*his2*	*DS/E*	*otr1R(Sph1)::ura4+*	*Phf1-myc::kanMX, Lsd1-FTP::NatN2*

S3 Table. List of strains used in this study (2 of 10)

**Strains (*S. pombe*)**	***mat***	***leu1***	***ade6***	***his2***	***ura4***	**Epigenetic Reporter**	**Mutation**
**SPFM607**	Mat1 Msmt0	*leu1-32*	*216*	*his2*	*DS/E*	*otr1R(Sph1)::ura4+*	*Phf1-myc::kanMX, Lsd1-FTP::NatN2*
**SPFM612**	Mat1 Msmt0	*leu1-32*	*216*	*his2*	*DS/E*	*otr1R(Sph1)::ura4+*	*Phf1-myc::kanMX, Lsd1-ΔHMG-FTP::NatN2*
**SPFM404**	h+	*leu1-32*	*216*	*plus*	*DS/E*	*otr1R(Sph1)::ura4+*	*Phf2-myc::kanMX*
**SPJC136**	Mat1 Msmt0	*leu1-32*	*210 or 216*	*his2*	*DS/E*	*otr1R(Sph1)::ura4+*	*Phf2-myc::kanMX, Lsd1-FTP::NatN2*
**SPJC154**	Mat1 Msmt0	*leu1-32*	*210 or 216*	*his2*	*DS/E*	*otr1R(Sph1)::ura4+*	*Phf2-myc::kanMX, Lsd1-ΔHMG-FTP::NatN2*
**SPJC138**	Mat1 Msmt0	*leu1-32*	*210 or 216*	*his2*	*DS/E*	*otr1R(Sph1)::ura4+*	*Phf2-myc::kanMX, Lsd1-FTP::NatN2*
**SPJC155**	Mat1 Msmt0	*leu1-32*	*210 or 216*	*his2*	*DS/E*	*otr1R(Sph1)::ura4+*	*Phf2-myc::kanMX, Lsd1-ΔHMG-FTP::NatN2*
**SPJC160**	Mat1 Msmt0	*leu1-32*	*210 or 216*	*his2*	*DS/E*	*otr1R(Sph1)::ura4+*	*Phf1-myc::kanMX, Lsd2-FTP::NatN2*
**SPJC78**	h+	*leu1-32*	*210 or 216*	*plus*	*DS/E*	*otr1R(Sph1)::ura4+*	*Phf1-myc::kanMX, lsd2-ΔC-FTP::NatN2*
**SPJC161**	Mat1 Msmt0	*leu1-32*	*210 or 216*	*his2*	*DS/E*	*otr1R(Sph1)::ura4+*	*Phf1-myc::kanMX, Lsd2-FTP::NatN2*
**SPJC79**	h+	*leu1-32*	*210 or 216*	*plus*	*DS/E*	*otr1R(Sph1)::ura4+*	*Phf1-myc::kanMX, lsd2-ΔC-FTP::NatN2*
**SPJC164**	Mat1 Msmt0	*leu1-32*	*210 or 216*	*his2*	*DS/E*	*otr1R(Sph1)::ura4+*	*Phf2-myc::kanMX, Lsd2-FTP::NatN2*

S3 Table. List of strains used in this study (3 of 10)

Strains (*S. pombe*)	*mat*	*leu1*	*ade6*	*his2*	*ura4*	Epigenetic Reporter	Mutation
**SPJC84**	*Mat1 Msmt0*	*leu1-32*	*210 or 216*	*his2*	*DS/E*	*otr1R(Sph1)::ura4+*	*Phf2-myc::kanMX, lsd2-ΔC-FTP::NatN2*
**SPJC165**	*Mat1 Msmt0*	*leu1-32*	*210 or 216*	*his2*	*DS/E*	*otr1R(Sph1)::ura4+*	*Phf2-myc::kanMX, Lsd2-FTP::NatN2*
**SPJC88**	*Mat1 Msmt0*	*leu1-32*	*210 or 216*	*his2*	*DS/E*	*otr1R(Sph1)::ura4+*	*Phf2-myc::kanMX, lsd2-ΔC-FTP::NatN2*
**SPJC82**	*Mat1 Msmt0*	*leu1-32*	*210 or 216*	*his2*	*DS/E*	*otr1R(Sph1)::ura4+*	*Phf1-myc::kanMX, lsd2-ΔC-FTP::NatN2*
**SPJC132**	*Mat1 Msmt0*	*leu1-32*	*210 or 216*	*his2*	*DS/E*	*otr1R(Sph1)::ura4+*	*Phf1-myc::kanMX, Lsd1-FTP::NatN2*
**SPJC146**	*Mat1 Msmt0*	*leu1-32*	*210 or 216*	*his2*	*DS/E*	*otr1R(Sph1)::ura4+*	*Phf1-myc::kanMX, Lsd1-ΔHMG-FTP::NatN2*
**SPG1407**	*h90*	*leu1-32*	*216*	*his2 or plus*	*DS/E*	-	*clr4Δ::kanMX*
**SPJT502**	*diploid*	*leu1-32//leu1-32*	*210//216*	*his2//plus*	*DS/E//DS/E*	*otr1R(Sph1)::ura4+//otr1R(Sph1)::ura4+*	*lsd1-ΔHMG-FTP::NatN2//lsd1-ΔHMG-FTP::NatN2*
**KZ569**	*Mat1 Msmt0*	*leu1-32*	*210*	*his2*	*DS/E*	*otr1R(Sph1)::ura4+*	*clr4Δ::kanMX*
**JT531**	*Mat1 Msmt0*	*leu1-32*	*210*	*plus*	*DS/E*	*otr1R(Sph1)::ura4+*	*clr4Δ::kanMX, lsd1-ΔHMG-FTP::NatN2*
**JT459**	*Mat1 Msmt0*	*leu1-32*	*210*	*his2*	*DS/E*	*otr1R(Sph1)::ura4+*	*set1Δ::kanMX*
**JT458**	*Mat1 Msmt0*	*leu1-32*	*210 or 216*	*his2*	*DS/E*	*otr1R(Sph1)::ura4+*	*set1Δ::kanMX, lsd1-C2-FTP::NatN2*

S3 Table. List of strains used in this study (4 of 10)

Strains (*S. pombe*)	*mat*	*leu1*	*ade6*	*his2*	*ura4*	Epigenetic Reporter	Mutation
**SPFM348**	*Mat1 Msmt0*	*leu1-32*	*210 or 216*	*his2*	*?*	*otr1R(Sph1)::ura4+?*	*Lsd1-FTP::NatN2, clr4Δ::kanMX*
**SPFM337**	*Mat1 Msmt0*	*leu1-32*	*210 or 216*	*his2*	*?*	*otr1R(Sph1)::ura4+?*	*Lsd1-FTP::NatN2, set1Δ::kanMX*
**SPFM335**	*Mat1 Msmt0*	*leu1-32*	*210 or 216*	*his2*	*?*	*otr1R(Sph1)::ura4+?*	*Lsd1-FTP::NatN2, clr4Δ::kanMX, set1Δ::kanMX*
**SPFM340**	*Mat1 Msmt0*	*leu1-32*	*210 or 216*	*his2*	*?*	*otr1R(Sph1)::ura4+?*	*Lsd2-FTP::NatN2, clr4Δ::kanMX*
**SPFM341**	*h+*	*leu1-32*	*210 or 216*	*plus*	*?*	*otr1R(Sph1)::ura4+?*	*Lsd2-FTP::NatN2, clr4Δ::kanMX, set1Δ::kanMX*
**SPFM344**	*h+*	*leu1-32*	*210 or 216*	*plus*	*?*	*otr1R(Sph1)::ura4+?*	*Lsd2-FTP::NatN2, set1Δ::kanMX*
**SPKZ165**	*Mat1 Msmt0*	*leu1-32*	*210*	*his2*	*DS/E*	*otr1R(Sph1)::ura4+*	*Raf2-myc::kanMX*
**SPFM614**	*h+*	*leu1-32*	*216*	*plus*	*DS/E*	*otr1R(Sph1)::ura4+*	*Raf2-myc::kanMX, Lsd1-FTP::NatN2*
**SPFM627**	*Mat1 Msmt0*	*leu1-32*	*216*	*his2*	*DS/E*	*otr1R(Sph1)::ura4+*	*Swd2-GFP-HA::kanMX*
**SPFM624**	*Mat1 Msmt0*	*leu1-32*	*216*	*his2*	*DS/E*	*otr1R(Sph1)::ura4+*	*Swd2-GFP-HA::kanMX, Lsd1-FTP::NatN2*
**SPHL152**	*Mat1 Msmt0*	*leu1-32*	*216*	*his2*	*DS/E or D18*	*otr1R(Sph1)::ura4+?*	*Swd2-GFP-HA::kanMX*
**SPHL151**	*Mat1 Msmt0*	*leu1-32*	*216*	*his2*	*DS/E or D18*	*otr1R(Sph1)::ura4+*	*Swd2-GFP-HA::kanMX, Lsd2-FTP::NatN2*
**SPHL158**	*Mat1 Msmt0*	*leu1-32*	*216*	*his2*	*DS/E*	*otr1R(Sph1)::ura4+?*	*Raf2-myc::kanMX*

S3 Table. List of strains used in this study (5 of 10)

Strains (*S. pombe*)	*mat*	*leu1*	*ade6*	*his2*	*ura4*	Epigenetic Reporter	Mutation
SPHL157	*h+*	*leu1-32*	*216*	*plus*	*DS/E*	*otr1R(Sph1)::ura4+*	*Raf2-myc::kanMX, Lsd2-FTP::NatN2*
SPJT696	*Mat1 Msmt0*	*leu1-32*	*216 or 210*	*his2*	*?*	*otr1R(Sph1)::ura4+?*	*Lsd1-FTP::NatN2, set1Δ::kanMX*
SPAA115	*Mat1 Msmt0*	*leu1-32*	*216*	*his2*	*DS/E or D18*	*otr1R(Sph1)::ura4+*	*Lsd1-FTP::NatN2, spp1Δ::kanMX*
SPAA79	*Mat1 Msmt0*	*leu1-32*	*216*	*his2*	*DS/E*	*otr1R(Sph1)::ura4+*	*swd1Δ::kanMX, Lsd1-FTP::NatN2*
SPAA68	*Mat1 Msmt0*	*leu1-32*	*216*	*his2*	*DS/E*	*otr1R(Sph1)::ura4+*	*Lsd1-FTP::NatN2, swd3Δ::kanMX*
SPJC53	*h+*	*leu1-32*	*210*	*plus*	*DS/E*	*otr1R(Sph1)::ura4+*	*Lsd1-FTP::NatN2, swd2Δ::kanMX*
SPJC36	*Mat1 Msmt0*	*leu1-32*	*216*	*his2*	*DS/E*	*otr1R(Sph1)::ura4+*	*Lsd1-FTP::NatN2, swd2Δ::kanMX*
SPJC7	*Mat1 Msmt0*	*leu1-32*	*216*	*his2*	*DS/E*	*otr1R(Sph1)::ura4+*	*Lsd1-FTP::NatN2, shg1Δ::kanMX*
SPJC9	*Mat1 Msmt0*	*leu1-32*	*216*	*his2*	*DS/E*	*otr1R(Sph1)::ura4+*	*Lsd1-FTP::NatN2, sdc1Δ::kanMX*
SPJT697	*h+*	*leu1-32*	*216 or 210*	*plus*	*?*	*otr1R(Sph1)::ura4+?*	*Lsd2-FTP::NatN2, set1Δ::kanMX*
SPAA121	*Mat1 Msmt0*	*leu1-32*	*210*	*his2*	*DS/E or D18*	*otr1R(Sph1)::ura4+*	*Lsd2-FTP::NatN2, spp1Δ::kanMX*
SPAA123	*Mat1 Msmt0*	*leu1-32*	*210*	*his2*	*DS/E or D18*	*otr1R(Sph1)::ura4+*	*Lsd2-FTP::NatN2, swd1Δ::kanMX*
SPJC44	*Mat1 Msmt0*	*leu1-32*	*210*	*his2*	*DS/E*	*otr1R(Sph1)::ura4+*	*Lsd2-FTP::NatN2, swd3Δ::kanMX*
SPJC51	*Mat1 Msmt0*	*leu1-32*	*216*	*his2*	*DS/E*	*otr1R(Sph1)::ura4+*	*Lsd2-FTP::NatN2, swd2Δ::kanMX*
SPJC48	*Mat1 Msmt0*	*leu1-32*	*210*	*his2*	*DS/E*	*otr1R(Sph1)::ura4+*	*Lsd2-FTP::NatN2, ash2Δ::kanMX*

S3 Table. List of strains used in this study (6 of 10)

Strains (*S. pombe*)	*mat*	*leu1*	*ade6*	*his2*	*ura4*	Epigenetic Reporter	Mutation
SPJC22	*Mat1 Msmt0*	*leu1-32*	*210 or 216*	*his2*	*DS/E*	*otr1R(Sph1)::ura4+*	*Lsd2-FTP::NatN2, shg1Δ::kanMX*
SPJC13	*Mat1 Msmt0*	*leu1-32*	*216*	*his2*	*DS/E*	*otr1R(Sph1)::ura4+*	*Lsd2-FTP::NatN2, sdc1Δ::kanMX*
SPJT692	*Mat1 Msmt0*	*leu1-32*	*216 or 210*	*his2*	*DS/E*	*otr1R(Sph1)::ura4+*	*Lsd1-FTP::NatN2, clr4Δ::kanMX*
SPUG236	*Mat1 Msmt0*	*leu1-32*	*216*	*his2*	*?*	*otr1R(Sph1)::ura4+*	*Lsd1-FTP::NatN2, raf1Δ::kanMX*
SPUG255	*Mat1 Msmt0*	*leu1-32*	*210*	*his2*	*DS/E*	*otr1R(Sph1)::ura4+*	*Lsd1-FTP::NatN2, rik1Δ::kanMX*
SPUG266	*Mat1 Msmt0*	*leu1-32*	*216*	*his2? his3 D1*	*?*	*otr1R(Sph1)::ura4+?*	*Lsd1-FTP::NatN2, raf2Δ::kanMX*
SPHL106a	*Mat1 Msmt0*	*leu1-32*	*?*	*his2*	*DS/E*	*otr1R(Sph1)::ura4+?*	*Lsd1-FTP::NatN2, cul4-1::kanMX*
SPJT694a	*h+*	*leu1-32*	*216 or 210*	*plus*	*DS/E*	*otr1R(Sph1)::ura4+*	*Lsd2-FTP::NatN2, clr4Δ::kanMX*
SPFM471	*Mat1 Msmt0*	*leu1-32*	*?*	*his2*	*DS/E*	*otr1R(Sph1)::ura4+*	*Lsd2-FTP::NatN2, raf1Δ::kanMX*
SPUG284	*Mat1 Msmt0*	*leu1-32*	*216*	*his2*	*DS/E*	*otr1R(Sph1)::ura4+?*	*Lsd2-FTP::NatN2, rik1Δ::kanMX*
SPUG279	*Mat1 Msmt0*	*leu1-32*	*210*	*his2*	*?*	*otr1R(Sph1)::ura4+?*	*Lsd2-FTP::NatN2, raf2Δ::kanMX*
SPFM374	*Mat1 Msmt0*	*leu1-32*	*210 or 216*	*his2*	*DS/E*	*otr1R(Sph1)::ura4+?*	*Lsd2-FTP::NatN2, cul4-1::kanMX*
SPUG311	*Mat1 Msmt0*	*leu1-32*	*210 or 216*	*his2*	*DS/E*	*otr1R(Sph1)::ura4+*	*ddb1Δ::NAT, Lsd1-FTP::NatN2*
SPHL136	*h+*	*leu1-32*	*210*	*plus*	*DS/E*	*otr1R(Sph1)::ura4+?*	*ddb1Δ::NAT, Lsd2-FTP::NatN2*

S3 Table. List of strains used in this study (7 of 10)

Strains (*S. pombe*)	*mat*	*leu1*	*ade6*	*his2*	*ura4*	Epigenetic Reporter	Mutation
**SPHL108a**	*h+*	*leu1-32*	?	*his2*	?	*otr1R(Sph1)::ura4+?*	*Lsd1-FTP::NatN2, mts2-1*
**SPHL109**	*Mat1 Msmt0*	*leu1-32*	?	*his2*	?	*otr1R(Sph1)::ura4+?*	*Lsd2-FTP::NatN2, mts2-1*
**SPHL119a**	*Mat1 Msmt0*	*leu1-32*	?	*his2*	?	*otr1R(Sph1)::ura4+?*	*Lsd1-FTP::NatN2, mts2-1, set1Δ::kanMX*
**SPHL126**	*Mat1 Msmt0*	*leu1-32*	*210*	*his2*	*DS/E*	*ura5-14, lys7-2?*	*FLAG(3X)-Set1*
**SPHL128**	*h+*	*leu1-32*	*210*	*plus*	*D18*	*otr1R(Sph1)::ura4+*	*FLAG(3X)-Set1, clr4Δ::kanMX,*
**SPHL149**	*Mat1 Msmt0*	*leu1-32*	*216*	*his2?*	?	*otr1R(Sph1)::ura4+?, ura5-14? lys7-2?*	*FLAG(3X)-Set1, raf1Δ::kanMX,*
**SPHL161**	*h+*	*leu1-32*	*216*	*plus*	?	*otr1R(Sph1)::ura4+?, ura5-14? lys7-2?*	*FLAG(3X)-Set1, raf2Δ::kanMX,*
**SPHL144**	*Mat1 Msmt0*	*leu1-32*	*216*	*his2*	?	*otr1R(Sph1)::ura4+?, ura5-14? lys7-2?*	*FLAG(3X)-Set1, rik1Δ::kanMX,*
**SPHL137**	*h+*	*leu1-32*	*216*	*plus*	*D18*	*otr1R(Sph1)::ura4+*	*FLAG(3X)-Set1, cul4-1::kanMX,*
**SPHL140**	*Mat1 Msmt0*	*leu1-32*	*210*	*his2*	*DS/E*	*otr1R(Sph1)::ura4+*	*FLAG(3X)-Set1, clr4W31G (sensitive to FOA)*
**SPHL155**	*Mat1 Msmt0*	*leu1-32*	*210*	*his2*	?	*otr1R(Sph1)::ura4+?, ura5-14? lys7-2?*	*FLAG(3X)-Set1, ddb1Δ::NatN2*
**SPHL240a**	*Mat1 Msmt0*	*leu1-32*	?	*his2*	?	*otr1R(Sph1)::ura4+?, ura5-14? lys7-2?*	*FLAG(3X)-Set1, brl1Δ::kanMX,*
**SPHL228a**	*Mat1 Msmt0*	*leu1-32*	?	*his2*	?	*otr1R(Sph1)::ura4+?, ura5-14? lys7-2?*	*FLAG(3X)-Set1, brl2Δ::kanMX,*
**SPHL202**	*Mat1 Msmt0*	*leu1-32*	*216*	*his2*	*DS/E*	*otr1R(Sph1)::ura4+*	*Lsd1-FTP::NatN2, brl1Δ::kanMX*
**SPHL206**	*h+*	*leu1-32*	*216*	*plus*	*DS/E*	*otr1R(Sph1)::ura4+*	*Lsd1-FTP::NatN2, brl2Δ::kanMX*
**SPHL208**	*Mat1 Msmt0*	*leu1-32*	*210*	*his2*	*DS/E*	*otr1R(Sph1)::ura4+?*	*Lsd2-FTP::NatN2, brl1Δ::kanMX*

S3 Table. List of strains used in this study (8 of 10)

Strains (*S. pombe*)	*mat*	*leu1*	*ade6*	*his2*	*ura4*	Epigenetic Reporter	Mutation
**SPHL212**	*h+*	*leu1-32*	*216*	*plus*	*DS/E*	*otr1R(Sph1)::ura4+?*	*Lsd2-FTP::NatN2, brl2Δ::kanMX*
**SPHL236**	*Mat1 Msmt0*	*leu1-32*	*210*	*his2*	*DS/E or D18*	-	*hht1/2/3 K4R, Lsd1-FTP::NatN2*
**SPHL237**	*Mat1 Msmt0*	*leu1-32*	*210*	*his2*	*DS/E or D18*	-	*hht1/2/3 K4R, Lsd2-FTP::NatN2*
**SPHL238**	*Mat1 Msmt0*	*leu1-32*	*210*	*his2*	*DS/E*	*otr1R(Sph1)::ura4+?*	*H2B-K119R(3X)FLAG::kanMX, Lsd1-FTP::NatN2*
**SPHL239**	*Mat1 Msmt0*	*leu1-32*	*210*	*his2*	*DS/E*	*otr1R(Sph1)::ura4+?*	*H2B-K119R(3X)FLAG::kanMX, Lsd2-FTP::NatN2*

Strains (*S. cerevisiae*)	Genotype	Reporters	Transformation Markers
**Y2H Gold**	*MATa, trp1-901, leu2-3, 112, ura3-52, his3-200, gal4Δ, gal80Δ, LYS2::GAL1UAS–Gal1TATA–His3, GAL2UAS–Gal2TATA–Ade2 URA3::MEL1UAS–Mel1TATA AUR1-C MEL1*	AbA^r, HIS3, ADE2, MEL1	*trp1, leu2*
**Y187**	*MATα, ura3-52, his3-200, ade2-101, trp1-901, leu2-3, 112, gal4Δ, gal80Δ, met–, URA3::GAL1UAS–Gal1TATA–LacZ, MEL1*	MEL1, LacZ	*trp1, leu2*
**Y2HGold [pGBKT7-53]**	The same genotype as Y2H gold, transformed pGBKT7-53 plasmid	AbA^r, HIS3, ADE2, MEL1	*trp1*
**Y187 [pGADT7- T]**	The same genotype as Y187, transformed pGADT7- T plasmid	MEL1, LacZ	*leu2*

S3 Table. A list of strains used in this study (9 of 10)

Strains (*S. cerevisiae*)	Genotype	Reporters	Transformation Markers
**Y2HGold [pGBKT7-Lam]**	The same genotype as Y2H gold+, transformed pGBKT7-Lam plasmid	AbA^r, HIS3, ADE2, MEL1	*trp1*
**Y2H 1**	The same genotype as Y2H gold+, transformed pGBKT7-Lsd1 plasmid	AbA^r, HIS3, ADE2, MEL1	*trp1*
**Y2H 2**	The same genotype as Y2H gold+, transformed pGBKT7-Lsd2 plasmid	AbA^r, HIS3, ADE2, MEL1	*trp1*
**Y2H 3**	The same genotype as Y2H gold+, transformed pGBKT7-Phf1 plasmid	AbA^r, HIS3, ADE2, MEL1	*trp1*
**Y2H 4**	The same genotype as Y2H gold+, transformed pGBKT7-Phf2 plasmid	AbA^r, HIS3, ADE2, MEL1	*trp1*
**Y2H 5**	The same genotype as Y187+, transformed pGADT7- Lsd1 plasmid	MEL1, LacZ	*leu2*
**Y2H 6**	The same genotype as Y187+, transformed pGADT7- Lsd2 plasmid	MEL1, LacZ	*leu2*
**Y2H 7**	The same genotype as Y187+, transformed pGADT7- Phf1 plasmid	MEL1, LacZ	*leu2*
**Y2H 8**	The same genotype as Y187+, transformed pGADT7- Phf2 plasmid	MEL1, LacZ	*leu2*
**Y2H 9 (Positive Control)**	Mating Y2HGold [pGBKT7-53] and Y187 [pGADT7- T]	AbA^r, HIS3, ADE2, MEL1	*trp1, leu2*
**Y2H 10 (Negative Control)**	Mating Y2HGold [pGBKT7-Lam] and Y187 [pGADT7- T]	AbA^r, HIS3, ADE2, MEL1	*trp1, leu2*
**Y2H 11**	Mating Y2H 1 and Y2H 5 (Lsd1-BD Lsd1-AD)	AbA^r, HIS3, ADE2, MEL1	*trp1, leu2*
**Y2H 12**	Mating Y2H 1 and Y2H 6 (Lsd1-BD Lsd2-AD)	AbA^r, HIS3, ADE2, MEL1	*trp1, leu2*
**Y2H 13**	Mating Y2H 1 and Y2H 7 (Lsd1-BD Phf1-AD)	AbA^r, HIS3, ADE2, MEL1	*trp1, leu2*
**Y2H 14**	Mating Y2H 1 and Y2H 8 (Lsd1-BD Phf2-AD)	AbA^r, HIS3, ADE2, MEL1	*trp1, leu2*

S3 Table. A list of strains used in this study (10 of 10)

Strains (*S. cerevisiae*)	Genotype	Reporters	Transformation Markers
**Y2H 15**	Mating Y2H 2 and Y2H 5 (Lsd2-BD Lsd1-AD)	AbA^r, HIS3, ADE2, MEL1	*trp1*, *leu2*
**Y2H 16**	Mating Y2H 2 and Y2H 6 (Lsd2-BD Lsd2-AD)	AbA^r, HIS3, ADE2, MEL1	*trp1*, *leu2*
**Y2H 17**	Mating Y2H 2 and Y2H 7 (Lsd2-BD Phf1-AD)	AbA^r, HIS3, ADE2, MEL1	*trp1*, *leu2*
**Y2H 18**	Mating Y2H 2 and Y2H 8 (Lsd2-BD Phf2-AD)	AbA^r, HIS3, ADE2, MEL1	*trp1*, *leu2*
**Y2H 19**	Mating Y2H 3 and Y2H 5 (Phf1-BD Lsd1-AD)	AbA^r, HIS3, ADE2, MEL1	*trp1*, *leu2*
**Y2H 20**	Mating Y2H 3 and Y2H 6 (Phf1-BD Lsd2-AD)	AbA^r, HIS3, ADE2, MEL1	*trp1*, *leu2*
**Y2H 21**	Mating Y2H 3 and Y2H 7 (Phf1-BD Phf1-AD)	AbA^r, HIS3, ADE2, MEL1	*trp1*, *leu2*
**Y2H 22**	Mating Y2H 3 and Y2H 8 (Phf1-BD Phf2-AD)	AbA^r, HIS3, ADE2, MEL1	*trp1*, *leu2*
**Y2H 23**	Mating Y2H 4 and Y2H 5 (Phf2-BD Lsd1-AD)	AbA^r, HIS3, ADE2, MEL1	*trp1*, *leu2*
**Y2H 24**	Mating Y2H 4 and Y2H 6 (Phf2-BD Lsd2-AD)	AbA^r, HIS3, ADE2, MEL1	*trp1*, *leu2*
**Y2H 25**	Mating Y2H 4 and Y2H 7 (Phf2-BD Lsd1-AD)	AbA^r, HIS3, ADE2, MEL1	*trp1*, *leu2*
**Y2H 26**	Mating Y2H 4 and Y2H 8 (Phf2-BD Phf2-AD)	AbA^r, HIS3, ADE2, MEL1	*trp1*, *leu2*
