# Supplementary material for: The Cross-Regulation Between Set1, Clr4, and Lsd1/2 in Schizosaccharomyces pombe
Source: PLoS Genet. 2024 Jan 5;20(1):e1011107. doi: 10.1371/journal.pgen.1011107 (PMC10795994; doi:10.1371/journal.pgen.1011107)
Supplement: S4 Table — (PDF) [file pgen.1011107.s014.pdf]

S4 Table. List of oligonucleotides used in this study (1 of 5)

Primer	Sequence	Purpose
**Kan3**	GCGCAATCACGAATGAATAA	KanMX6 genotyping
**Kan2**	ATCATTGGCAACGCTACCTT	KanMX6 genotyping
**NAT2**	GGTGTCGGTGGTGAAGGACC	NatN2 genotyping
**Nat3**	TGCCCTGCCCCTAATCTCGA	NatN2 genotyping
**Kan_Fwd**	CGGATCCCCGGGTTAATTAA	Amplify KanMX/NatN2 cassette
**Nat2 S2**	ATCGATGAATTCGAGCTCG	Amplify KanMX/NatN2 cassette
**Lsd1 RV2**	AAACCCATTTCTCCTAACCC	*lsd1-FTP::NatN2* genotyping
**Lsd2 RV4**	GAGTGAGAGTAGTAGCTGTG	*lsd2-FTP::NatN2* genotyping
**Lsd1ctd_checkF1**	CGTCTCCTGTTCCACCATCA	*lsd1-ΔHMG-FTP::NatN2* genotyping
**Lsd2ctd_checkF1**	GCTTGTAACTGAACTTCTTGG	*lsd2-ΔC-FTP::NatN2* genotyping
**Phf1 RV2**	GTAACACCCTGACATTGACC	*Phf1-myc::kanMX* genotyping
**Phf2 TRV2**	GACTTGGACCCTGTTGATAG	*Phf2-myc:kanMX* genotyping
**BC8D218C Prom1**	AATGGTTGGCAAAGGATGAG	*sah1*$^{+}$ promoter (3′ RV)
**BC8D218C Prom2**	TTGGCTGCATTTACTCACC	*sah1*$^{+}$ promoter (5′ FW)
**Lsd1 BD New FW**	GCATATGGCCATGGAGGCCGAATTCATGATGG ATTTGTCTTCGAAAGAT	Amplify Lsd1-BD cassette
**Lsd1 BD New RV**	GCGGCCGCTGCAGGTCGACGGATCCTCATGAG AAATTGTCAGGGAATAC	Amplify Lsd1-BD cassette
**Lsd1 AD New FW**	TATGGCCATGGAGGCCAGTGAATTCATGATGG ATTTGTCTTCGAAAGAT	Amplify Lsd1-AD cassette
**Lsd1 AD New RV**	TCTGCAGCTCGAGCTCGATGGATCCTCATGAGA AATTGTCAGGGAATAC	Amplify Lsd1-AD cassette

S4 Table. List of oligonucleotides used in this study (2 of 5)

Primer	Sequence	Purpose
**Lsd2 BD New FW**	CATATGGCCATGGAGGCCGAATTCATGAACA CGAGCGAGAATGATCC	Amplify Lsd2-BD cassette
**Lsd2 BD New RV**	GCGGCCGCTGCAGGTCGACGGATCCTTAATA TCGTGTTCCATTATAACC	Amplify Lsd2-BD cassette
**Lsd2 AD New FW**	ATGGCCATGGAGGCCAGTGAATTCATGAACA CGAGCGAGAATGATCC	Amplify Lsd2-AD cassette
**Lsd2 AD New RV**	TCTGCAGCTCGAGCTCGATGGATCCTTAATAT CGTGTTCCATTATAACC	Amplify Lsd2-AD cassette
**Phf1 BD FW**	GCATATGGCCATGGAGGCCGAATTCATGTCTC AAAAGAATTTTTTCGAC	Amplify Phf1-BD cassette
**Phf1 BD RV**	GCGGCCGCTGCAGGTCGACGGATCCTCATAA AACAGTAGCACATAAATT	Amplify Phf1-BD cassette
**Phf1 AD FW**	TATGGCCATGGAGGCCAGTGAATTCATGTCTC AAAAGAATTTTTTCGAC	Amplify Phf1-AD cassette
**Phf1 AD RV**	TCTGCAGCTCGAGCTCGATGGATCCTCATAAA ACAGTAGCACATAAATT	Amplify Phf1-AD cassette
**Phf2 BD FW1**	GCATATGGCCATGGAGGCCGAATTCATGCCG AATTCATCGTACTATG	Amplify Phf2-BD cassette fragment 1 (5′ FW)
**Phf2 BD RV2**	GCGGCCGCTGCAGGTCGACGGATCCTTAAAT ACTACTAAGAACAATTGG	Amplify Phf2-BD cassette fragment 2 (3′ RV)
**Phf2 AD FW1**	TATGGCCATGGAGGCCAGTGAATTCATGCCG AATTCATCGTACTATG	Amplify Phf2-AD cassette fragment 1 (5′ FW)
**Phf2 AD RV2**	TCTGCAGCTCGAGCTCGATGGATCCTTAAATA CTACTAAGAACAATTGG	Amplify Phf2-AD cassette fragment 2 (3′ RV)
**Phf2 FW2**	TAAATACTCGAATAGAAAAATT	Amplify Phf2-BD/Phf2-AD cassette fragment 2 (5′ FW)
**Phf2 RV1**	AATTTTTCTATTCGAGTATTTAGCTTGAATAG TGTTAGTGATA	Amplify Phf2-BD/Phf2-AD cassette fragment 2 (3′ RV)
**Raf2 RV2**	GATGATCGTAAATGACCTGAG	*Raf2-myc::kanMX* genotyping
**Clr4 RV3**	CTTCCTGATATAGCACTTCCC	*clr4Δ::kanMX* genotyping
**Set1 RV2**	ACTTCATTGATGGCTTCCCG	*set1Δ::kanMX* genotyping
**Spp1 FW1**	GTGGATTCAGAGACGATG	Amplify *spp1Δ::kanMX* cassette fragment 1 (5′ FW)
**Spp1 Kan RV**	TTAATTAACCCGGGGATCCGGCTTCCTTTTTT AACTCTTCC C	Amplify *spp1Δ::kanMX* cassette fragment 1 (3′ RV)
**Spp1 Kan FW**	CGAGCTCGAATTCATCGATGTCGTGTTTGTGT TCCCATAG	Amplify *spp1Δ::kanMX* cassette fragment 2 (5′ FW)
**Spp1 RV1**	CTGAACGATTTAAAGTACAGCG	Amplify *spp1Δ::kanMX* cassette fragment 2 (3′ RV)
**Spp1 RV2**	GTCAAAATAAAGCGTCTGAGC	*spp1Δ::kanMX* genotyping
**Swd1 RV2**	GAGTACTGTATGTGATGC	*swd1Δ::kanMX* genotyping

S4 Table. List of oligonucleotides used in this study (3 of 5)

Primer	Sequence	Purpose
**Swd3 FW1**	CCTTTGAACCACGACCTGC	Amplify *swd3Δ::kanMX* cassette fragment 1 (5′ FW)
**Swd3 Kan RV**	TTAATTAACCCGGGGATCCGGGGGAAACGCTG ACACATG	Amplify *swd3Δ::kanMX* cassette fragment 1 (3′ RV)
**Swd3 Kan FW**	CGAGCTCGAATTCATCGATCCAAATTACAGATC CGAGTAC	Amplify *swd3Δ::kanMX* cassette fragment 2 (5′ FW)
**Swd3 RV1**	CCAAACTCGTACATAAGGATC	Amplify *swd3Δ::kanMX* cassette fragment 2 (3′ RV)
**Swd3 RV2**	GCTTTCTCGTCAACCTGC	*swd3Δ::kanMX* genotyping
**Ash2 FW1**	GTGGATTCAGAGACGATG	Amplify *ash2Δ::kanMX* cassette fragment 1 (5′ FW)
**Ash2 Kan RV**	TTAATTAACCCGGGGATCCGCCCAAAGTAGCTT GCCAAG	Amplify *ash2Δ::kanMX* cassette fragment 1 (3′ RV)
**Ash2 Kan FW**	CGAGCTCGAATTCATCGATGATGACGGTTCTCT TGGT	Amplify *ash2Δ::kanMX* cassette fragment 2 (5′ FW)
**Ash2 RV1**	GTCACAGAAGACGTATTTGGG	Amplify *ash2Δ::kanMX* cassette fragment 2 (3′ RV)
**Ash2 RV2**	CGGACCTTGCTCATCTTGTC	*ash2Δ::kanMX* genotyping
**Shg1 FW1**	GCCTTAACAACAGAACATCG	Amplify *shg1Δ::kanMX* cassette fragment 1 (5′ FW)
**Shg1 Kan RV**	TTAATTAACCCGGGGATCCGGTGTTACATCCCT ATCCATG	Amplify *shg1Δ::kanMX* cassette fragment 1 (3′ RV)
**Shg1 Kan FW**	CGAGCTCGAATTCATCGATATGGATGGGCGCA AATGAAG	Amplify *shg1Δ::kanMX* cassette fragment 2 (5′ FW)
**Shg1 Rv1**	CAGCAGTAATAGAGATCCG	Amplify *shg1Δ::kanMX* cassette fragment 2 (3′ RV)
**Shg1 RV2**	CCTGATCTCTACATACTCTCAC	*shg1Δ::kanMX* genotyping
**Sdc1 FW1**	GTAAGCCCAGTTGTGAATGC	Amplify *sdc1Δ::kanMX* cassette fragment 1 (5′ FW)
**Sdc1 Kan RV**	TTAATTAACCCGGGGATCCGGTTTGACATAATT CAGGCG	Amplify *sdc1Δ::kanMX* cassette fragment 1 (3′ RV)
**Sdc1 Kan FW**	CGAGCTCGAATTCATCGATGCAGGTTCAAACGT CTTTTTC	Amplify *sdc1Δ::kanMX* cassette fragment 2 (5′ FW)
**Sdc1 RV1**	CAGTCGGCCAAGTATTATC	Amplify *sdc1Δ::kanMX* cassette fragment 2 (3′ RV)
**Sdc1 RV2**	CATCAGCAATCGGACGAAAC	*sdc1Δ::kanMX* genotyping
**Raf1 3' RV2**	TGGTTTTTGTTAACGGCATG	*raf1Δ::kanMX* genotyping
**Raf2 3' RV2**	GGTAATCGTCGCGTATGGTC	*raf2Δ::kanMX* genotyping
**Rik1 FW1**	GGTAGACACAGCGACTTCAT	Amplify *rik1Δ::kanMX* cassette fragment 1 (5′ FW)
**Rik1 Kan RV**	TTAATTAACCCGGGGATCCGCCGTAGAAAATA AAGGGATAGAGG	Amplify *rik1Δ::kanMX* cassette fragment 1 (3′ RV)

S4 Table. List of oligonucleotides used in this study (4 of 5)

Primer	Sequence	Purpose
**Rik1 Kan FW**	CGAGCTCGAATTCATCGATACGGAAATTGGTGCTATTGG	Amplify *rik1Δ::kanMX* cassette fragment 2 (5′ FW)
**Rik1 RV1**	CCATCAATAAACGGGACTGG	Amplify *rik1Δ::kanMX* cassette fragment 2 (3′ RV)
**Rik1 RV2**	TTTATCAGTGCCATGTTGCG	*rik1Δ::kanMX* genotyping
**Cul4 RV2**	CGGTACTTAGCGCGAACAC	*cul4-1::kanMX* genotyping
**Ddb1 FW1**	CACATACTACACCGCACCATG	Amplify *ddb1Δ::NatN2* cassette fragment 1 (5′ FW)
**Ddb1 Kan RV**	TTAATTAACCCGGGGATCCGCCACTATTACGTTCCAGGAG	Amplify *ddb1Δ::NatN2* cassette fragment 1 (3′ RV)
**Ddb1 Kan FW**	CGAGCTCGAATTCATCGATCGCAGCAGATTTAATGAAGTC	Amplify *ddb1Δ::NatN2* cassette fragment 2 (5′ FW)
**Ddb1 RV1**	GTTTTATCGACCGTGGAAAGAG	Amplify *ddb1Δ::NatN2* cassette fragment 2 (3′ RV)
**Ddb1 RV2**	CCAACACCACCATCTCCTAC	*ddb1Δ::NatN2* genotyping
**Set1p_ups Fwd**	GTCAAGGCTCATTATTGGAA	*Flag-Set1* genotyping
**FLAG-3x-N-ter R**	CTTGTCATCGTCATCCTTGTAATCGATGTCATGATCTTTATAATCACCGTCATGGTCTTTGTAGTC	*Flag-Set1* genotyping
**Cen_ura4_F**	GCCATGTCAGATTTGACACAACTC	*otr1R(Sph1)::ura4*$^{+}$ genotyping
**Cen_ura4_R**	CGTGAGTATACAAACAAATACACTAGG	*otr1R(Sph1)::ura4*$^{+}$ genotyping
**Ura5_orf68_F**	CCTTAAAATCAGGCAGAAAA	*ura5-14* genotyping
**Ura5-14_chk_R**	GCGATAATAGTCTCCTGCAA	*ura5-14* genotyping
**Brl1 Fw1**	GAAGAAGCCATTGTCGTTAG	Amplify *brl1Δ::kanMX* cassette fragment 1 (5′ FW)
**Brl1 Kan RV**	TTAATTAACCCGGGGATCCGCCTCGATTCATTCACAACGC	Amplify *brl1Δ::kanMX* cassette fragment 1 (3′ RV)
**Brl1 Kan FW**	CGAGCTCGAATTCATCGATAAGTACATGTCCGCAGTGTG	Amplify *brl1Δ::kanMX* cassette fragment 2 (5′ FW)
**Brl1 Rv1**	GGAGAGTGAGTCATGATGCTAG	Amplify *brl1Δ::kanMX* cassette fragment 2 (3′ RV)
**Geno Brl1 FW**	GAGGCTCAAGTATACAAAGG	*brl1Δ::kanMX* genotyping
**Geno Brl1 RV**	GCTAGTTACAGTCGCTACAG	*brl1Δ::kanMX* genotyping
**Brl2 Fw1**	GCCCTCTACCACTTCACTTTTACC	Amplify *brl2Δ::kanMX* cassette fragment 1 (5′ FW)
**Brl2 Kan RV**	TTAATTAACCCGGGGATCCGTAAGATTGTGGGGGCATCTG	Amplify *brl2Δ::kanMX* cassette fragment 1 (3′ RV)

S4 Table. List of oligonucleotides used in this study (5 of 5)

Primer	Sequence	Purpose
**Brl2 Kan FW**	CGAGCTCGAATTCATCGATGGTCGTGGATTTG GTGCTTC	Amplify *brl2Δ::kanMX* cassette fragment 2 (5′ FW)
**Brl2 Rv1**	GACGTTAAACAGATGGTCCG	Amplify *brl2Δ::kanMX* cassette fragment 2 (3′ RV)
**Geno Brl2 FW**	CTCTCTGTGGTCATGGCTTTTG	*brl2Δ::kanMX* genotyping
**Geno Brl2 RV**	GAATAGAGGCAGCTGTCTTC	*brl2Δ::kanMX* genotyping
**qLeu1F1**	ATTGCTCGTTTGGCTGCTTG	qPCR: *leu1*$^{+}$ (Endogenous Contorl)
**qLeu1R1**	TACAGTCTTGCGCCAAAGAC	qPCR: *leu1*$^{+}$ (Endogenous Contorl)
**q_cen(dg)_FOR**	AATTGTGGTGGTGTGGTAATAC	qPCR: Centromere (*dg*)
**q_cen(dg)_REV**	GGGTTCATCGTTTCCATTCAG	qPCR: Centromere (*dg*)
**q_mat_FOR**	GTCCGAGGCAATACAACTTTGG	qPCR: Mating-type locus (*cenH*)
**q_mat_REV**	GGTTGACAGTAGGAGATATTTACAG	qPCR: Mating-type locus (*cenH*)
**q_Lsd1-FW**	GGCAAAGAGGGAGAAAGAGA	qPCR: *lsd1*$^{+}$
**q_Lsd1-RV**	TTGATGGTGGAACAGGAGAC	qPCR: *lsd1*$^{+}$
**q_Lsd2-FW**	TTCAAATGCAGGTGTCGCTC	qPCR: *lsd2*$^{+}$
**q_Lsd2-FW**	ATTGCTGAGAAGACGGAGAG	qPCR: *lsd2*$^{+}$
**q_act FOR**	GGTTTCGCTGGAGATGATG	qPCR: *act1*$^{+}$ (Endogenous Contorl)
**q_act REV**	ATACCACGCTTGCTTTGAG	qPCR: *act1*$^{+}$ (Endogenous Contorl)
**Pan5 rtpcr FW**	AGAGAGACAGAGATTGATGC	qPCR: *pan5*$^{+}$ (Endogenous Contorl)
**Pan5 rtpcr RV**	TGCATTCATTGCAACCTTGG	qPCR: *pan5*$^{+}$ (Endogenous Contorl)
