# Supplementary material for: The Cross-Regulation Between Set1, Clr4, and Lsd1/2 in Schizosaccharomyces pombe
Source: PLoS Genet. 2024 Jan 5;20(1):e1011107. doi: 10.1371/journal.pgen.1011107 (PMC10795994; doi:10.1371/journal.pgen.1011107)
Supplement: Attachment — Submitted filename: Response to reviewers.pdf [file pgen.1011107.s022.pdf]

WAKE FOREST
UNIVERSITY

Ke Zhang Reid. Ph.D.
Associate Professor
Department of Biology
Wake Forest University
455 Vine Street
Winston Salem, NC 27101

PLOS Genetics Editor Office
Songtao Jia, Ph.D.
Guest Editor

Wendy Bickmore, Ph.D.
Section Editor

Dear Dr. Jia and Dr. Bickmore,

Please find enclosed our revised manuscript (PGENETICS-D-23-00858) entitled "The Cross-Regulation Between Set1, Clr4, and Lsd1/2 in *Schizosaccharomyces pombe*" by Liu *et al* to be considered for publication in PLOS Genetics. We appreciate your kind help in reviewing our manuscript. In response to insightful comments from the reviewers, we have revised the paper by performing additional experiments and modifying the text and figure legends. We are grateful for the improvements that have resulted from the useful comments of the reviewers. Below is our detailed response to specific comments by the reviewers, which are indicated in *italics*:

Reviewer #1: Liu et al. demonstrated the cross-regulation between histone-modifying enzymes. To my best knowledge, this cross-regulation has never been described before, and this finding will help understand how distinct posttranslational modifications of histones intimately play a role in shaping the transcriptome. However, at least to me, it is not clear enough how biologically significant the cross-regulation is. Also, some experiments need to be done to conclude.

We sincerely appreciate the reviewer's recognition of the novel cross-regulation we have unveiled among the three histone-modifying enzyme complexes in our manuscript. The insightful feedback on the need to elucidate the biological significance of this intricate interplay has been invaluable.

Initially, we were confounded by the interwoven dynamics between Set1, Clr4, and Lsd1/2. It is logical that Clr4 would destabilize Set1 or Lsd1/2 since these two enzymes typically oppose Clr4's function. However, the puzzling aspect is why Set1, a H3K4 methyltransferase, would enhance the stability of the Lsd complex, responsible for removing H3K4 di- and mono-methylation. While this relationship seems counterintuitive at first glance, the elevation of Lsd protein levels by Set1 could amplify Lsd activities,

which counteract Clr4's function and prevent its excessive activity. This complex relationship can be likened to the simple adage: "my enemy's enemy is my friend." The intricate cross-regulation among these enzymes ensures the fine-tuned activities, generating a flexible regulatory circuit that upholds cellular homeostasis.

In the context of gene expression, Clr4 plays a pivotal role in promoting gene silencing. Dysregulated gene silencing can trigger issues such as cellular senescence. Set1 and Lsd1/2, by counteracting Clr4, act as safeguards against uncontrolled senescence. However, the opposing roles of Set1 and Lsd1/2 also ensure that the degree of inhibition is not overly severe, preventing the other extreme of uncontrolled growth. The promotion of Lsd1/2 by Set1 fosters a balanced regulation, averting the dominance of one pathway over the other and thereby maintaining cellular homeostasis.

While the exact mechanisms underlying this cross-regulation remain unknown, we propose that this intricate relationship plays a crucial role in maintaining cellular homeostasis, enhancing precision in cellular processes, and contributing to the robustness and adaptability of biological systems. To underscore the biological significance of this relationship, we have incorporated additional sentences in the introduction and discussion sections of our revised manuscript.

The modified text can be found on lines 84-88 (Introduction) and 463-470 (Discussion).

(1) Based on the genetic interactions shown in Fig. 2A, I'd say that Lsd1 and Lsd2 primarily target H3K9me and H3K4me, respectively. If this description is correct, both clr4Δset1Δlsd2-ΔC and clr4Δset1Δlsd1-ΔHMG should be viable. I wonder if this speculation is correct. If clr4Δ or set1Δ fail to suppress the inviability of Δset1Δlsd2-ΔC or clr4Δlsd2-ΔC, respectively, I'd like to know the plausible reason why the triple mutants are not viable. I believe the additional genetic interaction analyses will further strengthen the conclusion.

We sincerely appreciate the insightful suggestion provided by the reviewer. In response, we conducted genetic crosses in an attempt to obtain clr4Δ set1Δ lsd2-ΔC and clr4Δ set1Δ lsd1-ΔHMG triple mutants. Our tetrad analysis unequivocally demonstrates the lethality of these triple mutants (S1D Fig). This critical finding underscores the complexity of the genetic relationship between Set1, Clr4, and Lsd1/2.

The lethality of the triple mutants is likely because Clr4 and Lsd2 exhibit essential overlapping functions on non-histone substrates, as do Set1 and Lsd1. While our statement that "Lsd1 and Lsd2 primarily target H3K9me and H3K4me, respectively" is based on published results [1-7], the genetic interactions (Fig 2) and this recent discovery collectively suggest that their roles extend beyond mere histone modification. It becomes increasingly evident that Set1, Clr4, and Lsd1/2 likely possess additional functions beyond histone modification. The observation aligns with the fact that mutations in all three copies of histone H3, resulting in H3K4A or H3K9A, do not induce lethality [8, 9]. In contrast, the deletion of Lsd1 leads to severe growth defects, and the

absence of Lsd2 is not viable. It is intriguing to note that Clr4 and Lsd1/2 proteins are absent in budding yeast, implying that the cross-regulated relationship among Set1, Clr4, and Lsd1/2 is likely to be indispensable and conserved in higher eukaryotes, which harbor heterochromatin. Our ongoing research endeavors are geared toward unveiling the non-histone substrates that may be subjected to cross-regulation by these protein-modifying enzymes, thus contributing to the fine-tuning of cellular homeostasis.

In accordance with the reviewer's suggestion, we have incorporated a supplemental figure (S1D Fig) and made the necessary textual modifications to emphasize these critical findings: lines 279-283.

(2) WB results such as Fig. 3A and 3B, there are more than one band of Lsd1 and Lsd2. These multiple bands are specific to tagged proteins, as untagged strain samples did not show any signal. What is the nature of those bands? Any posttranslational modifications?

That's an excellent question, and we appreciate your curiosity. To detect Lsd1-FTP or Lsd2-FTP in our western blot analysis, we employed Peroxidase-Anti-Peroxidase (PAP) antibody. This antibody has demonstrated its reliability by producing clean results with no detectable signal in untagged strain samples.

The presence of multiple bands in our western blot analysis (2A & 3B) likely represent various forms of tagged Lsd1/2 proteins, most of which may be degraded or post-transcriptionally modified. It is important to note that Lsd1/2 are inherently large and relatively unstable proteins. During the preparation of whole-cell extracts using the standard bead-homogenization technique, it is plausible that the large Lsd1/2 proteins are fragmented into distinct pieces, resulting in the appearance of multiple bands on the gel. Additionally, the observed bands could be indicative of the inherently unstable nature of Lsd1/2 proteins, which undergo rapid degradation during the sample preparation process. Lastly, we also cannot rule out the possibility that these bands represent poly-ubiquitylated forms of Lsd proteins, such as in Fig 4 A-F. We have added "" in the figures to indicate the bands that are likely to be full-length Lsd proteins.*

(3) The changes in the steady-state levels of Lsd1 and Lsd2 in mutants (e.g., clr4Δ and set1Δ) are clear, but have you tested transcription rate, mRNA levels, translation efficiency (or ribosome occupancy on mRNA), or protein stability? I suspect the changes result from the increased or decreased protein half-life, and I did not see such analyses (or did I miss them?!)

We conducted an RNA-seq analysis to investigate whether the deletion of Clr4 and Set1 had any impact on the mRNA levels of Lsd1 and Lsd2, as shown in the S9-10 (A-B) Figs. In addition, we performed qRT-PCR to validate that the changes in mRNA levels do not correspond with alterations in protein levels of Lsd1 and Lsd2 in the absence of Set1 or Clr4, as illustrated in the S9-10 (C-D) Figs. In alignment with the reviewer's observation, we concur that the protein stability (half-life) of Lsd1 and Lsd2 is

affected without Set1 or Clr4. This is supported by the notable alterations in the poly-ubiquitylation levels of Lsd proteins in the presence or absence of Set1 or Clr4, as depicted in Fig 4. Furthermore, our findings reveal that Lsd1-FTP protein levels are augmented at 33°C in the nonfunctional proteasome mst2-1 mutant. Importantly, the poly-ubiquitylation levels are not further elevated in the mts2-1 set1Δ mutant, signifying that Set1 plays a role in stabilizing the half-life of Lsd1 protein by safeguarding it from ubiquitin-mediated protein degradation. These results collectively highlight the intricate regulatory mechanisms governing the stability and degradation of Lsd1 and Lsd2 proteins in response to loss of Set1 and Clr4, as well as the proteasome machinery.

(4) I see the cross-regulation between the three protein complexes does exist. However, I am afraid I still do not quite understand the significance of the cross-regulation. What kind of advantage would the cross-regulation have? For example, can bulk histone methylation assessed by WB be maintained even in lsd mutants?

As previously discussed, the cross-regulation among these enzymes serves as an essential mechanism for fine-tuning their activities, culminating in the establishment of a flexible regulatory circuit that achieves cellular homeostasis. However, it's important to emphasize that this cross-regulation cannot be solely attributed to histone modifications. In the case of S. pombe, the bulk H3K9me is challenging to detect via western blotting, and the enrichment level can only be accurately assessed using techniques such as chromatin immunoprecipitation. Moreover, our previous findings reveal that while H3K9me2 levels increase in the catalytic mutants of lsd1-ao and lsd2-ao at heterochromatic regions, mutants like lsd1-ΔHMG and lsd2-ΔC lead to a decrease in H3K9me2 levels [10]. This result suggests the histone demethylase-independent roles of Lsd1 and Lsd2 in gene silencing within heterochromatin.

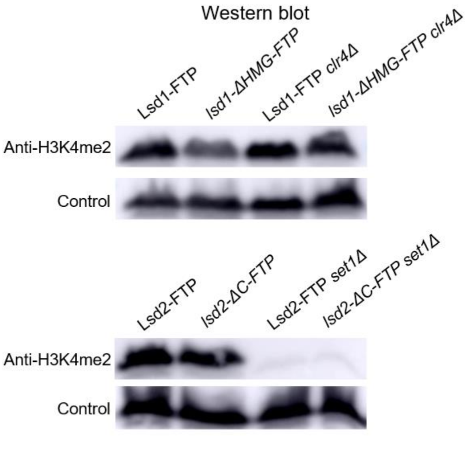

To further substantiate our findings, we conducted western blot analysis to investigate bulk H3K4me2 levels in lsd1-ΔHMG and lsd2-ΔC mutants (shown on the left). Our results indicate that lsd1-ΔHMG reduces bulk H3K4me2 levels, which is consistent with our prior discovery that Lsd1 has functions extending beyond removing histone K4/K9 methylations [10]. In the discussion section, we have extensively addressed the potential involvement of non-histone substrates modified by these protein-modifying enzymes, shedding light on their pivotal roles in the intricate cross-regulation. This discussion can be found on lines 554-568.

(5) As to the Y2H assays, was there any particular reason why you did not spot serial dilutions of budding yeast? The data shown in S3 Fig are qualitative, but data with the

serial dilutions are more quantitative. Or is qualitative data sufficient for your conclusion?

We appreciate the reviewer's suggestion. We performed a serial dilution assay for the Y2H assays. The conclusion agrees with Fig 1. Due to space limitations, we added the data to S5 Fig.

(6) line 101 and S Tables, "the 5' end" and "5' FW" should be "the 5´ end" and "5´ FW" Use "prime" not "apostrophe."

We thank the reviewer for carefully reviewing the manuscript. We have now fixed the typo: line 105, and S2 Table.

Reviewer #2: In this manuscript, Liu and colleagues investigate the crosstalk regulation between Set1 (H3K4methyltransferase), Clr4 (H3K9methyltransferase) and the lysine demethylases Lsd1 and Lsd2 in the model fission yeast. By performing genetic, biochemical and omics approaches the authors show that C-terminal truncations of Lsd1 and Lsd2 do not affect their complex composition but impair their chromatin binding, while also demonstrate that Clr4 and Set1 have opposite effects on Lsd1 and Lsd2 protein levels but not on transcripts. Importantly, such regulation is achieved through the ubiquitin-proteasome-dependent pathway. In addition, the authors show that Lsd1/2 protein levels increase upon heat stress and that Clr4 associated complex (CLRC) regulates Set1 levels. Overall, the data presented is convincing and increase our understanding of how lysine demethylases can be regulated. Please find below my comments which would improve this already fine manuscript.

_ While centromeric and mating-type silencing are readily detectable in the lsd1 and lsd2 mutant cells, it is not clear whether the mutant Lsd1 and Lsd2 proteins still localize to such regions. The authors shall consider including these data. I also wonder whether the upregulation at cenH can be noticeable in cells harboring Kint2:ura4+ marker or, a lternatively, mat2P upregulation (i.e. haploid meiosis) in REIIΔ cells.

We greatly appreciate the insightful suggestion made by the reviewer. As a response, we have revisited both the RNA-seq and ChIP-seq data, and we have included a S2 Fig to address the reviewer's comments. In light of this, we have also modified the text from line 215 to 220: "When comparing wild-type Lsd1 and Lsd2 proteins, we found lower enrichments at these constitutive heterochromatic regions in contrast to their higher enrichments at promoter regions (S2 Fig and Fig 1C). However, the enrichments of lsd1 and lsd2 C-terminal mutant proteins exhibited a modest increase at specific loci associated with the loss of silencing, in accordance with the reported roles of Lsd proteins in heterochromatic silencing", as reported previously from our group [10].

In our analysis, we did not observe the upregulation of cenH region, and the strains we used for the ChIP assay have no Kint2::ura4^{+} marker but are REIIΔ cells. Interestingly,

we did observe that the mat2 regions are upregulated in lsd1 C-terminal mutant cells from the RNA-seq data. In addition, lsd1 mutant protein enrichments are slightly enhanced in ChIP-Seq data in REIIΔ cells. The data is included in S2 Fig.

_ Discussion: Despite the fact that PLoS Genetics does not have length restriction, discussion is well written but it seems to be very long for the standard reader and therefore it should be shorten.

We appreciate this suggestion, and we have shortened the discussion.

_ By performing Western blots, the authors clearly show that Clr4 and Set1 modulate the protein levels of Lsd1 and Lsd2 in opposite ways. It would be nice to demonstrate such regulation by employing a microscopical approach (e.g. GFP-tagging Lsd1 and Lsd2). This is not compulsory, but it would improve the ms.

We highly appreciate your insightful suggestion. In response to this, we performed imaging experiments to observe Lsd1- and Lsd2-GFP proteins within clr4Δ and set1Δ genetic backgrounds, maintaining the standard temperature of 30°C. Our results indicate a notable decrease in the signal intensities of both Lsd1- and Lsd2-GFP in the set1Δ background, in comparison to the wild-type. Conversely, in the clr4Δ background, although there is a visibly increase in Lsd1- and Lsd2-GFP signal intensities, this change does not reach statistical significance when compared with the wild-type. These findings have been added as S8 Fig. Additionally, we have amended the manuscript to reflect these findings, specifically modifying the text from lines 320-322.

_ Y2H experiments: The authors state "Lsd2 does not physically interact with Lsd1, Phf1, or Phf2". I guess the authors mean direct interaction as Co-IP clearly shows such interactions. In addition, for the technical reasons explained in Fig S3 there is no available data for Lsd2-Phf1 interaction.

The reviewer's observation is accurate. In response, we have updated the model (Fig 1L & Fig 7) and revised the text to reflect that we were unable to reach a definitive conclusion regarding the Lsd2-Phf1 interaction. Line: 160, 245, 266-267.

_ Fig. 2G-H and lines 295-298: The commented differences for Lsd1 and Lsd2 enrichments are not robust. Can the authors show a couple of specific examples that illustrate this point?

Your suggestion is highly valuable. To illustrate this point, we have incorporated S6 and S7 Figs (line 313), which focus on two specific loci from each of the three chromosomes.

Minor comments

Fig. 1H: It is not clear what antibody was used in ChIP.

We employed an anti-Myc antibody for ChIP in Fig 1H. In Fig 1 legend (lines 1441-1442) and the methods section (lines 659-665), we detailed our adapted ChIP protocol, which involved enriching Myc-tagged Phf proteins in the FTP background. This was achieved through a pre-clearing step that utilized TEV cleavage and overnight incubation with IgG beads before the addition of the anti-Myc antibody. Notably, the pre-clearing procedure was highly effective, as we did not observe a substantial signal in Lsd-FTP strains lacking Myc-tagged Phf1 or Phf2.

S2 Fig: Nomenclature (i.e. Lsd1+, Lsd2+)

We have modified figures for correct nomenclature.

Line 142: Typo "In S. Pombe"

We corrected it. Line:146.

Line 243: Define the product of Ade2 (i.e. Phosphoribosylaminoimidazole carboxylase, involved in adenine biosynthesis)

We defined it as suggested (lines 254-255).

Lines 263-265: Based on the data shown, it looks like set1∆ is epistatic to lsd2-∆C. So, I am not sure if this can be defined as a positive interaction.

We defined positive interaction because lsd2-∆C suppresses the silencing defect of set1∆ at the mating type locus (S1 Fig).

Line 401: Typo "ubiqitin"

We fixed typo.

Reviewer #3: Histone-modifying enzymes play critical roles in gene expression and chromatin organization. In fission yeast, Set1 is the sole H3K4 methyltransferase while Clr4 is the H3K9 methyltransferase. In addition, fission yeast has also two highly conserved H3K4 and H3K9 demethylases, Lsd1 and Lsd2. In this manuscript, the authors found that Lsd1/2 interacts with the Clr4 complex (CLRC) and Set1-associated complexes (COMPASS). The further showed that Clr4 and Set1 modulate the protein levels of Lsd1 and Lsd2 in opposite ways through the ubiquitin-dependent pathway. In addition, the authors showed that Set1 mediates Lsd1/2 level during heat stress. The

results of this work are interesting. The experiments in the manuscript are generally well designed and executed. However, there are some concerns, especially regarding evidence to support the conclusion, as listed below:

Major

1. To convincingly show that Clr4 and Set1 mediate Lsd1 and Lsd2 through the ubiquitin-dependent pathway, the in vitro ubiquitination assays are needed.

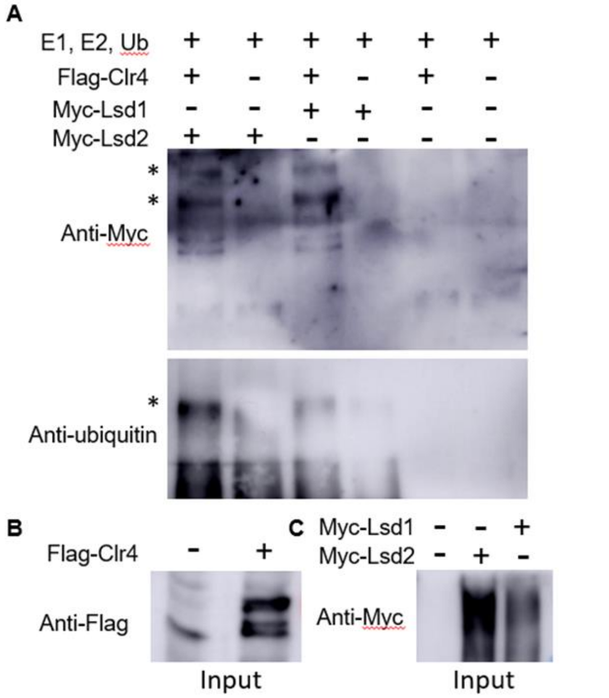

S Fig. CLRC ubiquitylates recombinant Lsd1 and Lsd2 proteins *in vitro*.
(A) Cell extracts from *S. pombe* with Flag-Clr4 and *S. cerevisiae* with Myc-Lsd1 or Myc-Lsd2 were examined via western blot using indicated antibodies. (B) Ubiquitylation assays conducted *in vitro* used enriched Flag-Clr4 as the E3 enzyme and enriched Myc-Lsd1 and Myc-Lsd2 as substrates, with western blot analysis using indicated antibodies identifying ubiquitylated Lsd species, marked by asterisks.

We appreciate this valuable suggestion. COMPASS lacks E3 ubiquitin ligase activity. So, we conducted an in vitro ubiquitination assay with CLRC as the enzyme. Flag-Clr4 was isolated from S. pombe using anti-Flag conjugated agarose, serving as the E3 enzyme, while Myc-Lsd1 and Myc-Lsd2, produced in budding yeast, were enriched with anti-Myc conjugated agarose. The essential components—E1, E2, and Ubiquitin—were sourced from the E3 Ligase Auto-Ubiquitylation Assay Kit (ab139469). Our experimental procedure closely followed the kit's guidelines, with a notable modification: both the enzyme and the substrates reacted on agarose beads.

Initially, we intended to use a different in vitro ubiquitination assay kit (BML-UW0970-0001) from Enzo Life Science, preferred for its enhanced activity. However, due to a backorder delaying its availability until early December, it fell outside our revision timeline. Despite this setback, our results, though not optimal, promisingly demonstrate that CLRC can ubiquitinate Lsd1 and Lsd2 in vitro. This finding aligns with our in vivo assays illustrated in Figure 4. Due to the data quality, we are hesitant to include the data in the manuscript. The result is shown on the left. Our future investigations will be dedicated to identifying the precise lysine residues that may be targeted by both E3 ubiquitin ligases and methyltransferases, both in vitro and in vivo.

2. Assays in Fig 4S should be repeated to show any significant difference between WT and the mutants. Alternatively, RT-qPCR can be used.

We appreciate this valuable suggestion, which aligns with feedback from other reviewers. In response, we conducted qRT-PCR and have included the results in S9-10 Figs. The outcomes of these experiments clearly demonstrate that the observed variations in protein levels cannot be attributed to changes in mRNA levels (lines 324-327; 525-528). These results substantiate our overarching conclusion that the differences in protein levels between the wild-type and mutant Lsd strains are primarily governed by post-transcriptional regulatory mechanisms.

3. Line 365-367, Figure 2C and 2D: without Set1, Lsd1/2 protein levels are lowly expressed during heat stress. But at 30°C, Lsd1 in the set1 mutant is already very low. The authors may want to check if Lsd1 transcription is affected in the mutant by RT-qPCR.

As per the reviewer's valuable suggestion, we conducted qRT-PCR to examine the mRNA levels of Lsd1 in the presence and absence of Set1. The findings unequivocally indicate that alterations of the mRNA levels of Lsd1, as well as Lsd2, are not aligned with changes in their protein levels in cells lacking Set1(lines 377-380, S10 Fig). Once again, these results align with our overarching conclusion that the disparities in protein levels between wild-type and Set1 deletion cells are primarily governed by post-transcriptional regulatory mechanisms.

4. Line 374-375: “we only observed a slight decrease in ubiquitination of Lsd1and Lsd2 in the clr4∆ background”. I don’t find a slight decrease in ubiquitination of Lsd2. In my view, it is even a little bit of an increase from the figure.

	Protein intensity of Lsd1-FTP	intensity of Lsd1-FTP ubiquitin signals	ubiquitin signals/protein intensity	Ralative abandance (Mut/WT)
Wildtype	8276.255	8063.205	0.974257681	1
Clr4 deletion	12123.083	5001.941	0.412596449	0.423498277
Set1 deletion	1567.355	15410.82	9.832373649	10.0921695

	intensity of Lsd2-FTP	intensity of Lsd2-FTP ubiquitin signals	ubiquitin signals/protein intensity	Ralative abandance (Mut/WT)
Wildtype	7774.255	2761.527	0.355214358	1
Clr4 deletion	11835.497	3358.305	0.283748541	0.798809322
Set1 deletion	887.799	15501.477	17.46057047	49.15502447

		Relative Ubi abundance (Mut/WT)
Lsd1	WT	1
Lsd1	clr4Δ	0.423498277
Lsd1	set1Δ	10.0921695
Lsd2	WT	1
Lsd2	clr4Δ	0.798809322
Lsd2	set1Δ	49.15502447

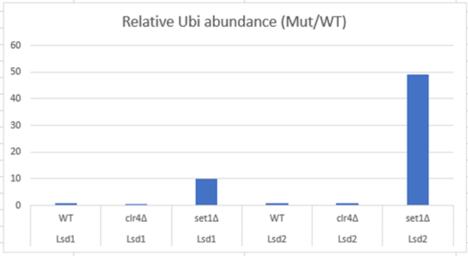

We apologize for any previous confusion and would like to offer additional clarification regarding our methodology and findings. Our band intensity quantification was conducted using Image J, and the resulting data has been consolidated in the Excel sheet presented on the left.

In the Lsd2 dataset, the ubiquitination signals in the clr4∆ sample are noticeably higher, primarily attributable to elevated protein levels in the pull-downed sample. Upon calculating the relative abundance of ubiquitin signals versus the amount of proteins, it becomes clear that the deletion of Clr4 leads to a reduction in ubiquitination levels for both Lsd1 and Lsd2. These quantification results further

strengthen our conclusion of a minor decrease in the ubiquitination of Lsd1 and Lsd2 in the clr4∆ background.

5. Line 238-247: the Y2H figures need more controls. Firstly, the cells grown on SD-LT plates indicating both plasmids have been transformed to the cells is necessary. In addition, the negative control of each BD or AD-containing protein with another empty plasmid are necessary in the same figure to show there is no self-activation in the Y2H assay.

We are grateful for this suggestion. We have remade those figures with all the controls that the reviewer requested, included in the S4 A-B Fig.

6. In some WB figures, like fig. 2c, fig.4 there are multiple bands. Please label the right one in the figure, so the readers can know them easily.

We utilized Peroxidase-Anti-Peroxidase (PAP) Antibody in our western blot analysis to detect Lsd1-FTP or Lsd2-FTP. This antibody is proven to be highly specific, as it exhibits no detectable signal in untagged strain samples. The presence of multiple bands in the western blot results can be attributed to tagged Lsd1/2 proteins, with a likelihood that some of them are in their truncated forms, such as Fig 3A-B. Lsd1 and Lsd2 are characterized by their substantial size and inherent instability. When we prepare whole cell extracts through standard bead-homogenization methods, it is plausible that these large proteins may be fragmented into various pieces, thus manifesting as multiple bands on the gel. Alternatively, these observed bands might also indicate the fragile nature of Lsd1/2 proteins, which could be susceptible to rapid degradation during the sample preparation process. It is important to note that we cannot rule out that these bands represent poly-ubiquitylated forms of Lsd proteins, such as Fig 4 A-F, although their sizes mostly appear smaller than those of the full-length Lsd proteins. Nevertheless, we have added "" in the figures to indicate the bands that are likely full-length Lsd proteins, as the reviewer suggested.*

Minor:

Line 142: change "S. Pombe" to "S. pombe".

We fixed it.

Line 174: please indicate which region is the amine oxidase domain located?

We added the information showing in lines 176-178.

Line 177: Please briefly explain the function of HMG domain.

We added a sentence at line 182 "a High Mobility Group domain implicated in DNA binding" to explain the function of the HMG domain.

Fig. 2I & 3: Please indicate which protein do you use as control in the fig? In addition, it indicates the Lsd1 and Lsd2 bands in the Fig. 2I.

We have the information for the loading control in the figure legends and we added "" indicating the full-length Lsd1 or Lsd2.*

Line 383: change "wildtype" to "wild-type".

We changed all "wildtype" to "wild-type".

In summary, we have addressed all the points raised by the reviewers. We look forward to hearing from you regarding the suitability of our paper for publication in PLOS. Genetics.

Sincerely,

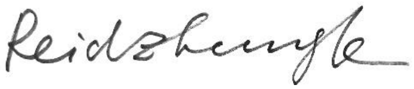

Ke Zhang Reid

References:

1. Shi Y, Lan F, Matson C, Mulligan P, Whetstine JR, Cole PA, et al. Histone demethylation mediated by the nuclear amine oxidase homolog LSD1. Cell. 2004;119(7):941-53. doi: 10.1016/j.cell.2004.12.012. PubMed PMID: 15620353.
2. Fang R, Barbera AJ, Xu Y, Rutenberg M, Leonor T, Bi Q, et al. Human LSD2/KDM1b/AOF1 regulates gene transcription by modulating intragenic H3K4me2 methylation. Mol Cell. 2010;39(2):222-33. doi: 10.1016/j.molcel.2010.07.008. PubMed PMID: 20670891; PubMed Central PMCID: PMCPMC3518444.
3. Fang R, Chen F, Dong Z, Hu D, Barbera AJ, Clark EA, et al. LSD2/KDM1B and its cofactor NPAC/GLYR1 endow a structural and molecular model for regulation of H3K4 demethylation. Mol Cell. 2013;49(3):558-70. Epub 20121220. doi: 10.1016/j.molcel.2012.11.019. PubMed PMID: 23260659; PubMed Central PMCID: PMCPMC3625064.
4. Nicolas E, Lee MG, Hakimi MA, Cam HP, Grewal SI, Shiekhattar R. Fission yeast homologs of human histone H3 lysine 4 demethylase regulate a common set of genes with diverse functions. J Biol Chem. 2006;281(47):35983-8. Epub 20060921. doi: 10.1074/jbc.M606349200. PubMed PMID: 16990277.
5. Opel M, Lando D, Bonilla C, Trewick SC, Boukaba A, Walfridsson J, et al. Genome-wide studies of histone demethylation catalysed by the fission yeast homologues of mammalian LSD1. PLoS One. 2007;2(4):e386. Epub 20070418. doi: 10.1371/journal.pone.0000386. PubMed PMID: 17440621; PubMed Central PMCID: PMCPMC1849891.

6. Lan F, Zaratiegui M, Villén J, Vaughn MW, Verdel A, Huarte M, et al. S. pombe LSD1 homologs regulate heterochromatin propagation and euchromatic gene transcription. Mol Cell. 2007;26(1):89-101. doi: 10.1016/j.molcel.2007.02.023. PubMed PMID: 17434129.
7. Gordon M, Holt DG, Panigrahi A, Wilhelm BT, Erdjument-Bromage H, Tempst P, et al. Genome-wide dynamics of SAPHIRE, an essential complex for gene activation and chromatin boundaries. Mol Cell Biol. 2007;27(11):4058-69. Epub 20070319. doi: 10.1128/mcb.02044-06. PubMed PMID: 17371846; PubMed Central PMCID: PMCPMC1900013.
8. Mikheyeva IV, Grady PJ, Tamburini FB, Lorenz DR, Cam HP. Multifaceted genome control by Set1 Dependent and Independent of H3K4 methylation and the Set1C/COMPASS complex. PLoS Genet. 2014;10(10):e1004740. Epub 20141030. doi: 10.1371/journal.pgen.1004740. PubMed PMID: 25356590; PubMed Central PMCID: PMCPMC4214589.
9. Okita AK, Zafar F, Su J, Weerasekara D, Kajitani T, Takahashi TS, et al. Heterochromatin suppresses gross chromosomal rearrangements at centromeres by repressing Tfs1/TFIIS-dependent transcription. Commun Biol. 2019;2:17. Epub 20190111. doi: 10.1038/s42003-018-0251-z. PubMed PMID: 30652128; PubMed Central PMCID: PMCPMC6329695.
10. Marayati BF, Tucker JF, Cerda DA, Hou TC, Chen R, Sugiyama T, et al. The Catalytic-Dependent and -Independent Roles of Lsd1 and Lsd2 Lysine Demethylases in Heterochromatin Formation in Schizosaccharomyces pombe. Cells. 2020;9(4). Epub 20200413. doi: 10.3390/cells9040955. PubMed PMID: 32295063; PubMed Central PMCID: PMCPMC7226997.
