# Supplementary material for: The Cross-Regulation Between Set1, Clr4, and Lsd1/2 in Schizosaccharomyces pombe
Source: PLoS Genet. 2024 Jan 5;20(1):e1011107. doi: 10.1371/journal.pgen.1011107 (PMC10795994; doi:10.1371/journal.pgen.1011107)
Supplement: Attachment — Submitted filename: Response to reviewers_12_4_2023.pdf [file pgen.1011107.s023.pdf]

WAKE FOREST
UNIVERSITY

Ke Zhang Reid. Ph.D.
Associate Professor
Department of Biology
Wake Forest University
455 Vine Street
Winston Salem, NC 27101

PLOS Genetics Editor Office
Songtao Jia, Ph.D.
Guest Editor

Wendy Bickmore, Ph.D.
Section Editor

Dear Dr. Jia and Dr. Bickmore,

Enclosed you will find our updated manuscript (PGENETICS-D-23-00858), titled "The Cross-Regulation Between Set1, Clr4, and Lsd1/2 in *Schizosaccharomyces pombe*" by Liu *et al*, submitted for consideration in PLOS Genetics. We are thankful for your assistance in the review process. Following the constructive feedback from the reviewers, we have enhanced the manuscript through revisions to the text, figures, and figure captions. The insightful suggestions from the reviewers have significantly contributed to the improvement of our manuscript.

In this resubmission, we have also uploaded a Striking Image with a corresponding caption to accompany our manuscript for consideration.

Below is our detailed response to specific comments by the reviewers and a list of additional changes, which are indicated in *italics*:

Reviewer #1: The authors satisfactorily addressed the comments I raised. This manuscript by Liu et al. should be accepted, and I expect to read a follow-up work on non-histone substrates.

We extend our sincere gratitude to Reviewer #1 for the endorsement of our manuscript's publication. As we progress, we are diligently developing our subsequent manuscript, which will specifically concentrate on non-histone substrates.

Reviewer #3: The authors addressed most of concerns. The only remaining major concern is the in vitro ubiquitination assays. The quality of the in vitro ubiquitination data that the authors provided is poor; in addition, the figure legend is confusing: does figure (A) show just a western blot or ubiquitination assays? Furthermore, I cannot find the figure legend for figure (C). Unless the authors can provide more convincing data, at minimum, they should acknowledge the possibility that the effect of CLRC on Lsd1/2 proteins may be indirect in the manuscript.

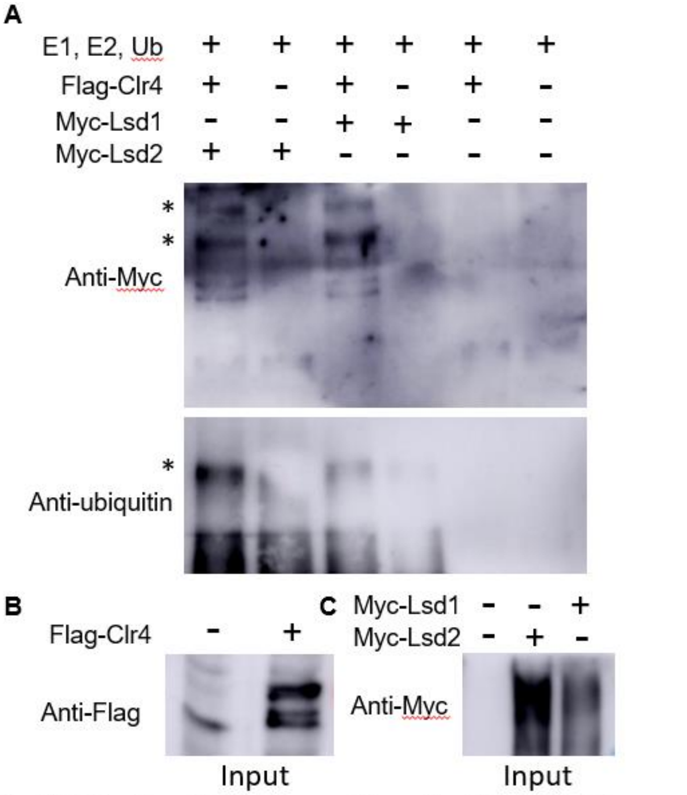

S Fig. CLRC ubiquitylates recombinant Lsd1 and Lsd2 proteins *in vitro*.
(A) Ubiquitylation assays conducted *in vitro* used enriched Flag-Clr4 from *S. pombe* as the E3 enzyme and enriched Myc-Lsd1 and Myc-Lsd2 from *S. cerevisiae* as substrates, with western blot analysis using indicated antibodies identifying ubiquitylated Lsd species, marked by asterisks. (B-C) Cell extracts from *S. pombe* with Flag-Clr4 (B) and *S. cerevisiae* with Myc-Lsd1 or Myc-Lsd2 (C) were examined via western blot using indicated antibodies.

We regret any confusion caused by our initial presentation of the in vitro ubiquitination assay. To clarify, we have amended the figure legend, and the revised figure can now be found on the left. We acknowledge the high cost and limited usage scope of the in vitro ubiquitination kit, which allows for only a single experiment with all necessary controls. Our use of Myc-Lsd1 and Myc-Lsd2, each bearing a single Myc tag, has resulted in a lower yield of purified protein. To enhance the experiment's quality, we are considering the use of either a 3X Myc tag or alternative tags for Lsd1 or Lsd2. While our current results are promising, we must convey our inability to produce a higher-quality figure within the 30-day timeframe. Our ongoing research will further explore the direct ubiquitylation of CLRC on Set1 and the Lsd1/2 complexes.

In line with the reviewer's insightful feedback, we have incorporated the suggestion of an indirect influence of CLRC on Lsd1/2 proteins into our manuscript. This acknowledgment is detailed on page 26, lines 577-578: "This suggests that Lsd1/2 are the likely targets of the Cul4-Rik1-Raf1 complex", lines 580-587: "While we are not certain that CLRC directly affects Lsd1/2 stability, our findings suggest that losing Clr4 or Raf2 disassembles CLRC, which likely impairs the Cul4-Rik1-Raf1 complex, potentially blocking the ubiquitination and subsequent degradation of Lsd1/2". Moreover, our model posits that "CLRC, whether directly or indirectly, influences the

protein levels of Lsd1/2 and Set1, potentially via the Cul4-Rik1-Raf1 E3 ubiquitin ligase complex," as stated on page 29, lines 640-641.

Minor:

About the Y2H in Figure 1J and 1K, the new version includes proper controls, which clarified my concerns. The minor point is that they didn't mention what plates they used; they just labeled +x-a-gal and -Adenine in the figure. Please provide more detailed information about the medium (is it -his, -Leu, -Trp?)

This point is indeed of critical significance. We clarify that all media utilized in Y2H experiments were devoid of leucine and tryptophan. Furthermore, the notation '+x-a-gal' indicates the inclusion of x-a-gal in the medium, while '-Adenine' signifies the absence of adenine. We have accordingly updated Fig. 1 and Supplementary Figures 4-5, along with their respective legends (lines 1451-1452, lines 1563-1564, and lines 1570-1571), to reflect this information more accurately. We are thankful for this valuable suggestion, as it has greatly enhanced the clarity of our presentation.

Additional changes made:

Line 200: Add a space between “HMGB1” and “structurally”.

Line 201: Change “tower” to “Tower”

Line 480: change “exhibits” to “exhibit”

Line 497: Change “Fig 1J-K” to “Fig 1J-K, S4-5 Figs”.

Line 521: Change “Fig 2G-H” to “Fig 2G-H, S6-7 Figs”.

We look forward to hearing from you regarding the suitability of our paper for publication in PLOS. Genetics.

Sincerely,

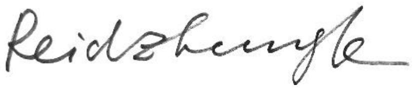

Ke Zhang Reid
